# Conserved immunomodulation and variation in host association by Xanthomonadales commensals in *Arabidopsis* root microbiota

**DOI:** 10.1038/s41477-025-01918-w

**Published:** 2025-02-19

**Authors:** Jana Ordon, Elke Logemann, Louis-Philippe Maier, Tak Lee, Eik Dahms, Anniek Oosterwijk, Jose Flores-Uribe, Shingo Miyauchi, Lucas Paoli, Sara Christina Stolze, Hirofumi Nakagami, Georg Felix, Ruben Garrido-Oter, Ka-Wai Ma, Paul Schulze-Lefert

**Affiliations:** 1 Department of Plant Microbe Interactions, Max Planck Institute for Plant Breeding Research, Cologne, Germany; 2 Center for Plant Molecular Biology, University Tuebingen, Tuebingen, Germany; 3 Regional Computing Centre, University of Cologne, Cologne, Germany; 4 Laboratory of Plant Physiology, Wageningen University and Research, Wageningen, the Netherlands; 5 Cluster of Excellence on Plant Sciences, Max Planck Institute for Plant Breeding Research, Cologne, Germany; 6 Department of Biology, Institute of Microbiology and Swiss Institute of Bioinformatics, ETH Zurich, Zurich, Switzerland; 7 Protein Mass Spectrometry Group, Max Planck Institute for Plant Breeding Research, Cologne, Germany; 8 Earlham Institute, Norwich Research Park, Norwich, UK; 9 Present Address: Department of Plant and Microbial Biology, University of Zurich, Zurich, Switzerland; 10 Present Address: Department of Plant Molecular Biology, University of Lausanne, Lausanne, Switzerland; 11 Present Address: Okinawa Institute of Science and Technology Graduate University, Okinawa, Japan; 12 Present Address: Global Health Institute, School of Life Sciences, École Polytechnique Fédérale de Lausanne, Lausanne, Switzerland; 13 Present Address: Institute of Plant and Microbial Biology, Academia Sinica, Taipei, Taiwan

**Keywords:** Microbe, Pattern recognition receptors in plants, Plant molecular biology, Microbiology

## Abstract

Suppression of chronic *Arabidopsis* immune responses is a widespread but typically strain-specific trait across the major bacterial lineages of the plant microbiota. We show by phylogenetic analysis and *in planta* associations with representative strains that immunomodulation is a highly conserved, ancestral trait across Xanthomonadales, and preceded specialization of some of these bacteria as host-adapted pathogens. *Rhodanobacter* R179 activates immune responses, yet root transcriptomics suggest this commensal evades host immune perception upon prolonged association. R179 camouflage likely results from combined activities of two transporter complexes (*dssAB*) and the selective elimination of immunogenic peptides derived from all partners. The ability of R179 to mask itself and other commensals from the plant immune system is consistent with a convergence of distinct root transcriptomes triggered by immunosuppressive or non-suppressive synthetic microbiota upon R179 co-inoculation. Immunomodulation through *dssAB* provided R179 with a competitive advantage in synthetic communities in the root compartment. We propose that extensive immunomodulation by Xanthomonadales is related to their adaptation to terrestrial habitats and might have contributed to variation in strain-specific root association, which together accounts for their prominent role in plant microbiota establishment.

## Abstract (web summary)

The authors show that immunosuppression is highly conserved in the bacterial order Xanthomonadales. This feature, which preceded their specialization as host-adapted pathogens, probably contributes to their prominence as core members of the plant microbiota.

## Main

Soil is one of the most species-rich environments^1^, and plants growing in this natural substrate are constantly in contact with a high density and diversity of microorganisms. Soil-dwelling microorganisms constitute the principal inoculum of the plant microbiota—that is, the entire community of plant-inhabiting microorganisms, which consists of only a fraction of the soil biome. Despite substantial variation between soil biomes, a similar taxonomic structure is found in the bacterial plant microbiota on roots and leaves, which is dominated by Proteobacteria, followed by Actinobacteria, Bacteroidetes and Firmicutes, collectively termed the core microbiota^2–7^. Photoassimilates produced by plant hosts are consumed during microbiota establishment and support heterotrophic microbial growth throughout the host’s lifetime^8,9^. In return, members of the bacterial plant microbiota improve plant performance by mobilizing mineral nutrients from soil for root uptake^10,11^, provide pathogen protection in roots and leaves^12–15^ and increase abiotic stress tolerance^16,17^. Numerous host-derived specialized metabolites or defence phytohormones such as salicylic acid, as well as bacterial substrate preferences for root-secreted molecules, influence root microbiota profiles at lower taxonomic ranks^18–24^.

The proliferation of invading, pathogenic microorganisms in plants is controlled by a two-tiered innate immune system, with immune responses triggered by families of cell-surface and intracellular immune receptors^25^. The presence of microbe-associated molecular patterns (MAMPs), which are often conserved among widely related taxa, and host-released damage-associated molecular patterns (DAMPs) are perceived by plasma-membrane-resident pattern recognition receptors (PRRs) in the extracellular space^26^. The majority of characterized PRRs belong to the families of leucine-rich repeat (LRR)-containing receptor-like kinases (RLKs) or LRR-containing receptor-like proteins (RLPs); the latter are characterized by the absence of a cytoplasmic kinase domain and constitutive interaction with the kinase SOBIR1 (refs. ^27,28^). Upon extracellular binding of the respective MAMP or DAMP, most characterized LRR-RLKs form heterodimers with the co-receptor BAK1 (ref. ^29^). Pattern-triggered immunity (PTI) is associated with a stereotypic array of defence-associated responses that ultimately restrict pathogen proliferation, including Ca^2+^ influx transients^30^, extensive transcriptional reprogramming^31^, the biosynthesis of the phytohormone ethylene^32^, NADPH-oxidase (for example, RBOHD)-dependent spikes in reactive oxygen species^33,34^, callose deposition^35^ and metabolic changes^36^. In axenic *Arabidopsis*, chronic exposure to immunogenic peptides such as flg22 (a bacterial peptide derived from flagellin that is perceived by the LRR-RLK FLS2) results in a dose-dependent reduction in plant growth^35^. This is interpreted as a trade-off, in which the host allocates its limited resources towards defence or growth^37^. Host-adapted phytopathogenic bacteria suppress PRR-triggered immune signalling by delivering typically strain-specific effectors into plant cells via the bacterial type III secretion system (T3SS)^38^. Intracellular immune receptors either bind to or detect the activity of these pathogen effectors, inducing effector-triggered immunity^39^.

Higher-order PRR mutants and defence phytohormone mutants in *Arabidopsis* exhibit altered bacterial community composition^19,40^. An intact PTI contributes to preventing *Arabidopsis* phyllosphere dysbiosis characterized by a higher bacterial load and a shift of the endophytic community^41^. This dysbiosis requires not only genetic depletion of multiple PRR complexes but also the MIN7 vesicle trafficking pathway or a specific mutation in a membrane-attack-complex/perforin-domain protein. Experiments with root commensals, including strains of a culture collection derived from *Arabidopsis thaliana* grown in Cologne agricultural soil (*At*-SPHERE)^5^, have demonstrated that interference with PTI outputs induced by exogenous application of synthetic flg22 is taxonomically widespread but typically strain-specific within a phylogenetic lineage^42–44^. In roots, the induction of PTI by treatment with flg22 alters the profile of synthetic communities (SynComs) containing only commensal strains that do not interfere with PTI, whereas the presence of immunomodulatory strains attenuates this effect^44^. SynComs consisting of immunomodulatory strains specifically downregulate a subset of immune-related genes^43,44^. However, our understanding of the mechanisms by which members of the microbiota interfere with PTI remains fragmentary^45^. While most commensal bacteria encode flagellin isoforms whose flg22 peptides evade detection by FLS2, others have evolved peptide variants that inhibit FLS2 activation through partial or complete antagonism^46,47^. The rhizobacteria *Pseudomonas capeferrum* WCS358 and *Pseudomonas simiae* WCS417 lower environmental pH through the production of gluconic acid and its derivative keto-gluconic acid and through amino acid biosynthesis pathways, respectively, resulting in reduced flg22-induced defence marker gene expression^42,48,49^. Type II secretion system (T2SS) mutants of the *Dyella japonica* MF79 commensal are impaired in suppressing PTI-associated defence marker gene expression in roots following acute flg22 treatment, while its T3SS mutants retain wild-type-like immunomodulatory activity^43^. However, the causative effectors exported through the T2SS of *D. japonica* and their host targets remain undefined.

In terrestrial environments, Xanthomonadales constitute a core bacterial order in the microbiota of photoautotrophic unicellular and multicellular eukaryotes^50,51^. The order is classified into Xanthomonadaceae and Rhodanobacteraceae and comprises both commensal and pathogenic strains^52^. The genus *Xanthomonas* consists mostly of pathogens infecting various aboveground plant organs, and these cause systemic vascular and local non-vascular diseases in nearly 400 plant species, with vascular infection probably being the ancestral mechanism^53,54^.

Using phylogenetic analysis of more than 1,700 Xanthomonadales genomes and strains of microbiota culture collections isolated from diverse photoautotrophic hosts, we show here that immunomodulation by Xanthomonadales is a conserved, ancestral trait that preceded their specialization as host-adapted plant pathogens. We demonstrate that *Arabidopsis* perceives the presence of *Rhodanobacter* R179, a root commensal belonging to the deepest branch of the Xanthomonadales, by detecting at least three immunogenic elicitors through the redundant activity of the immune modules EFR and SOBIR1. However, analyses of root transcriptomes and PTI-associated responses show that R179 hides itself and other commensals from host recognition upon prolonged root colonization. We generated and screened a mini-*Tn5* mutant library of *Rhodanobacter* R179 and identified commensal defence suppression system (*dss*) genes needed for immunomodulation and microbiota establishment in planta. A further mechanism of R179 camouflage involves clearance of extracellular immunogenic peptides by secreted peptidase(s), which probably serves a function in hiding the producer and other microbiota members from host recognition. We discuss the idea that the conservation of immunomodulation by xanthomonads might be related to their adaptation to terrestrial habitats and, together with strain-specific diversification to colonize roots, explains why they represent a core lineage of the plant microbiota.

## Results

### Immunomodulation is conserved in Xanthomonadales

**Fig. 1:**
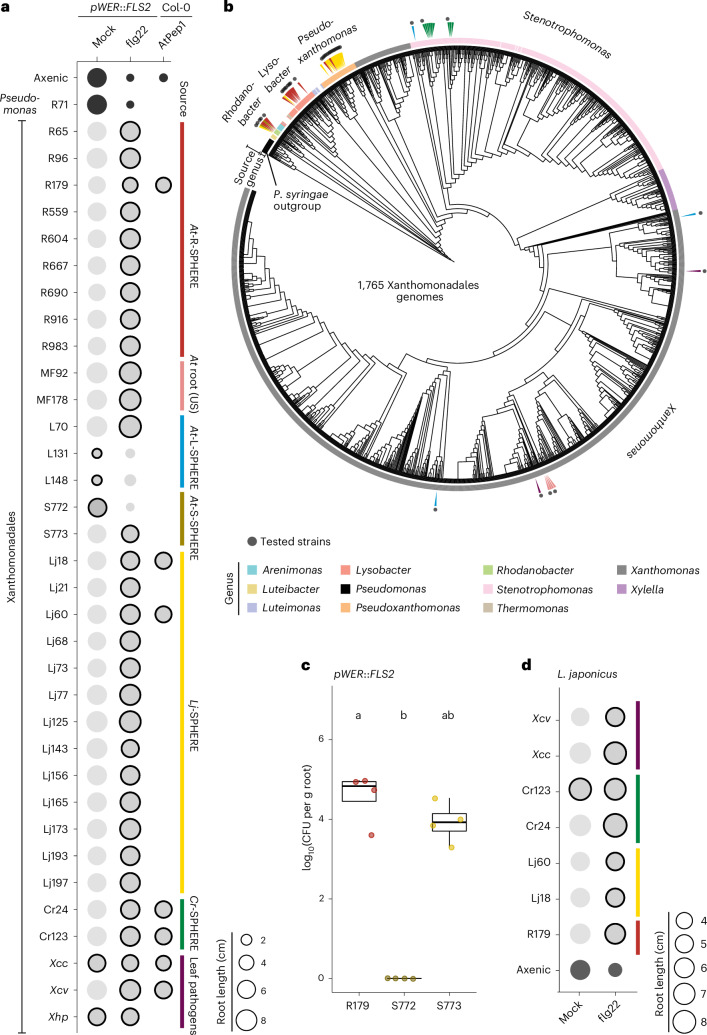
Phylogenetically diverse commensal and pathogenic Xanthomonadales strains suppress MAMP/DAMP-induced RGI in *A. thaliana* and *L. japonicus.* **a**, The sizes of the circles represent the median root length of *Arabidopsis pWER*::*FLS2–GFP* and wild-type Col-0 plants 3 weeks after co-treatment with mock, 1 µM flg22 or 1 µM AtPep1 (*x* axis) and individual Xanthomonadales strains from the *At*-R-SPHERE, *At*-L-SPHERE and *At*-S-SPHERE microbiota culture collections. The colours on the right indicate the sources of the strains. All strains were inoculated with start inocula OD_600_ = 0.0005. The black outlines indicate statistically significant differences compared with the corresponding axenic control (two-sided Dunn’s test: *P* < 0.001; adjusted by Bonferroni correction of *P*). All isolates were tested in at least three independent experiments (*n* > 3). **b**, Rooted phylogenetic tree constructed from 1,765 publicly available Xanthomonadales genomes. The tested strains are indicated with dots in the outer ring, and those from the culture collections are indicated by triangles in the second ring. The tree was constructed on the basis of 31 AMPHORA genes and rooted by an outgroup comprising 20 *Pseudomonas* genomes. Genera are indicated by colour in the inner ring. **c**, Bacterial titre represented as CFU of the *Arabidopsis* root-derived *Rhodanobacter* R179 and two soil-derived *Rhodanobacter*, S772 and S773, on *pWER*::*FLS2–GFP* roots after 2 weeks of co-cultivation in mono-associations (start inocula OD_600_ = 0.0005). The letters indicate statistical significance determined using a two-sided Dunn’s test (*P* < 0.05; adjusted by Bonferroni correction of *P*). *n* = 4 per *Rhodanobacter* strain. *n* = 4 biological samples per strain of one experiment representative of three experiments. In the box plots, the horizontal line indicates the median, the boxes extend to the 25th and 75th percentiles and the whiskers extend to the furthest point within 1.5× the interquartile range. **d**, The sizes of the circles represent the median root length of *L. japonicus* Gifu plants after 2 weeks of treatment with mock or 1 µM flg22 (*x* axis) and co-cultivation with a subset of the tested Xanthomonadales strains in **a** (the coloured bars represent different sources as in **a**; start inocula OD_600_ = 0.0005). The outlines indicate statistically significant differences compared with the corresponding axenic treatment group (two-sided Dunn’s test: *P* < 0.05; adjusted by Bonferroni correction of *P*). The experiment was repeated two times (*n* = 2). Source data

All 12 Xanthomonadales commensals of the *At*-R-SPHERE culture collection alleviate root growth inhibition (RGI) induced by flg22 (representative strains are shown in Fig. 1a)^5,44^. To determine whether the suppression of this immune response extends to Xanthomonadales strains derived from *Lotus japonicus* roots, the soil-borne unicellular alga *Chlamydomonas reinhardtii* or *Arabidopsis* grown in a US soil with a different inoculum source, we tested 17 additional isolates for their ability to suppress flg22-induced RGI in the *Arabidopsis* line *fls2/pWER*::*FLS2–GFP*, overexpressing *FLS2* in the root epidermis (*pWER*::*FLS2*; Fig. 1a)^50,55–57^. These 17 isolates alleviated flg22-induced RGI to similar degrees within 3 weeks of co-cultivation upon direct germination of *Arabidopsis* seeds on agar matrix containing flg22 (Fig. 1a).

**Extended Data Fig. 1:**
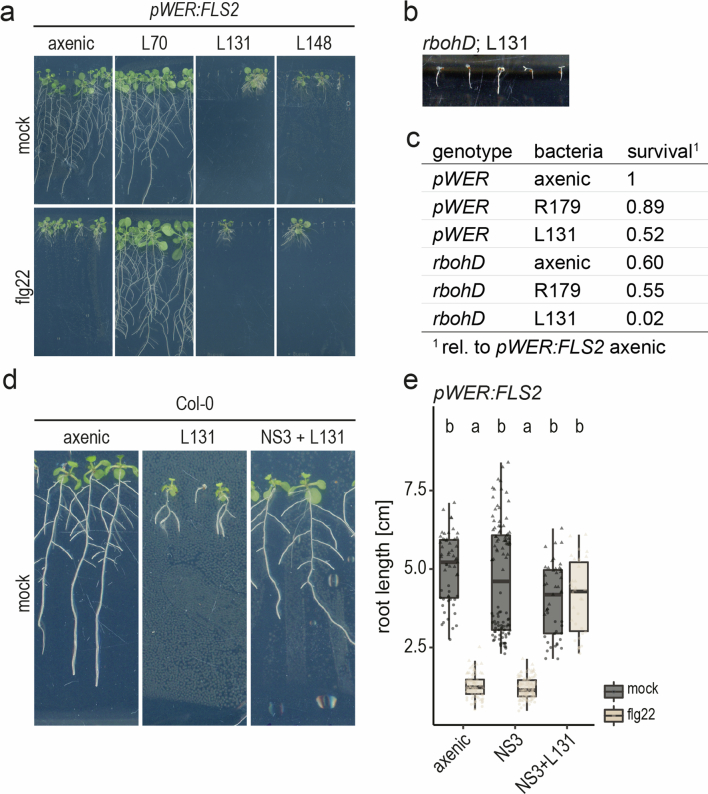
Detrimental activities of the leaf-derived *Xanthomonas* L131 can be uncoupled from the suppression of flg22-induced root growth inhibition. **a**, Phenotypes of three-week-old *Arabidopsis pWER:FLS2-GFP* plants cultivated with leaf-derived bacteria and 1 µM flg22 (start inocula OD_600_ = 0.0005). **b**, Phenotype of two-week-old *Arabidopsis rbohD* mutant plants cultivated in the presence of L131 (start inocula OD_600_ = 0.0005). **c**, Survival rate of *Arabidopsis pWER:FLS2-GFP* and *rbohD* mutant plants after two weeks of co-cultivation with the indicated bacterium compared to axenically grown plants. **d**, Phenotypes of two-week-old *Arabidopsis* Col-0 plants cultivated with the leaf-derived L131 together with a five-member non-immunosuppressive SynCom 3 (start inocula of each strain OD_600_ = 0.0005). **e**, Root length of *Arabidopsis pWER:FLS2-GFP* plants upon two weeks treatment with 1 µM flg22 (light brown) and co-cultivation with the leaf-derived L131 and a five-member non-immunosuppressive SynCom (start inocula of each strain OD_600_ = 0.0005). Shapes indicate two replicates with in total n = 57, 83, 104, 104, 47 and 34 measured primary roots of individual plants (from left to right). Letters indicate statistically significant differences determined using two-sided Dunn’s test (*p* < 0.05; adjusted by Bonferroni correction of *P*). The box plots center on the median and extend to the 25th and 75th percentiles, and the whiskers extend to the furthest point within 1.5× the interquartile range. A subset of the data has been shown in Fig. 2d. Source data

Three phytopathogenic strains, *Xanthomonas campestris* pv. *vesicatoria* (*Xcv* 85-10), *Xanthomonas campestris* pv. *campestris* (*Xcc* 8004) and *Xanthomonas hortorum* pv. *pelargonii* (*Xhp*), likewise alleviated flg22-induced RGI (Fig. 1a). *Xcc* and *Xhp* moderately inhibited root growth even in the absence of flg22 in our experimental setup (Fig. 1a). This growth inhibition was more pronounced with two leaf-derived *Xanthomonas* species, L131 and L148 (Fig. 1a and Extended Data Fig. 1a,b), recently described as opportunistic pathogens on immunocompromised *Arabidopsis rbohD* mutant plants^5,40,58,59^. Furthermore, L131 and L148 impaired seed germination (Extended Data Fig. 1c). This suggests that their pathogenic potential is not limited to leaves. Reduced seed germination and root growth were retained upon flg22 co-application, indicating that flg22-induced PTI does not protect the host against the detrimental impact of these opportunistic pathogens. However, L131-mediated detrimental activities were abolished by co-inoculation with a non-immunosuppressive SynCom consisting of five phylogenetically diverse *Arabidopsis*-root-derived bacteria that do not alleviate flg22-induced RGI (NS3 SynCom; Extended Data Fig. 1d and Supplementary Table 1)^44^, similar to a previously tested SynCom comprising 136 leaf-derived commensal bacteria^40^. Notably, co-inoculation of L131 with the NS3 SynCom but not the NS3 SynCom alone alleviated flg22-induced RGI (Extended Data Fig. 1e). Collectively, this indicates that the detrimental activities of L131 and the suppression of MAMP-induced RGI are independent traits.

We constructed a rooted phylogenetic tree comprising 1,765 publicly available Xanthomonadales draft genomes, including the genomes of our microbiota culture collections from *A. thaliana*, *L. japonicus* and *C. reinhardtii* (Fig. 1b and Supplementary Table 2). This revealed a global overrepresentation of phytopathogenic *Xanthomonas* species, particularly *Xcv*, *Xcc* and *Xanthomonas oryzae* pv. *oryzae* strains, as well as the opportunistic human pathogen *Stenotrophomonas maltophilia*. Strains isolated from *A. thaliana*, *L. japonicus* and *C. reinhardtii*, each cultured in the same Cologne agricultural soil, predominantly belong to *Rhodanobacter* and *Lysobacter*, *Pseudoxanthomonas* and *Stenotrophomonas*, respectively, indicating a potential preference of different Xanthomonadales genera for colonizing these three photoautotrophic hosts in Cologne agricultural soil (Fig. 1b). The strains tested for suppression of flg22-induced RGI belong to 7 of the 11 Xanthomonadales genera, each represented by at least three genomes (Fig. 1b). The suppression of immunity-associated RGI is thus highly conserved among Xanthomonadales regardless of the host species, organs or soil types from where they originate. Considering that both commensal and phytopathogenic Xanthomonadales can alleviate MAMP-induced RGI, this activity could be an ancestral trait that preceded specialization within Xanthomonadales to become plant-adapted pathogens. An exception to the conserved suppression of RGI was found in one of the two abundant soil-derived Xanthomonadales tested^5^, *Rhodanobacter* S772, which we failed to recover from 14-day-old inoculated roots (Fig. 1a,c). This suggests that the colonization of roots by Xanthomonadales is needed to relieve MAMP-induced RGI and that there is natural variation among soil-borne members of the Xanthomonadales in their ability to colonize *Arabidopsis* roots.

We next sought to explore the distribution of Xanthomonadales species across different environments (soil, aquatic, plant-associated and animal-associated). To that end, we leveraged the Microbe Atlas Project (MAP), which compiles over two million globally distributed 16S rRNA profiles. Xanthomonadales reference species (*n* = 57) were largely enriched in soil and plant-associated environments (78.9% of the samples, median across species; Methods). We also explored the distribution of potentially uncultivated Xanthomonadales across large-scale ocean, lake, animal-associated and soil metagenome-assembled genome (MAG) datasets, as well as a smaller plant-associated metagenomic survey. We found a total of 620 Xanthomonadales MAGs spanning 374 species in a global soil metagenomic dataset (40,039 MAGs spanning 21,076 species; 1.5% and 1.8%, respectively)^60^ and 110 Xanthomonadales MAGs out of 910 in total (12.1%) in a metagenomic study of leafy greens^61^. By contrast, we did not identify any genome belonging to the Xanthomonadales order in a continent-scale lake metagenomic dataset (1,184 MAGs spanning 1,008 species)^62^ or a rumen-associated metagenomic dataset (4,941 genomes)^63^, while we found only 69 Xanthomonadales MAGs from 10 species in an integrated human gut dataset (287,002 MAGs spanning 4,645 species; 0.02% and 0.2%, respectively)^64^ and 7 Xanthomonadales genomes from 6 species in a global open-ocean dataset (26,309 MAGs spanning 5,098 species; 0.03% and 0.1%, respectively)^65^. Taken together, these results provide evidence that terrestrial environments are the main reservoir for Xanthomonadales and suggest that soil, soil-dwelling algae and flowering plants may be the primary natural habitats for this entire order of Gammaproteobacteria.

**Fig. 2:**
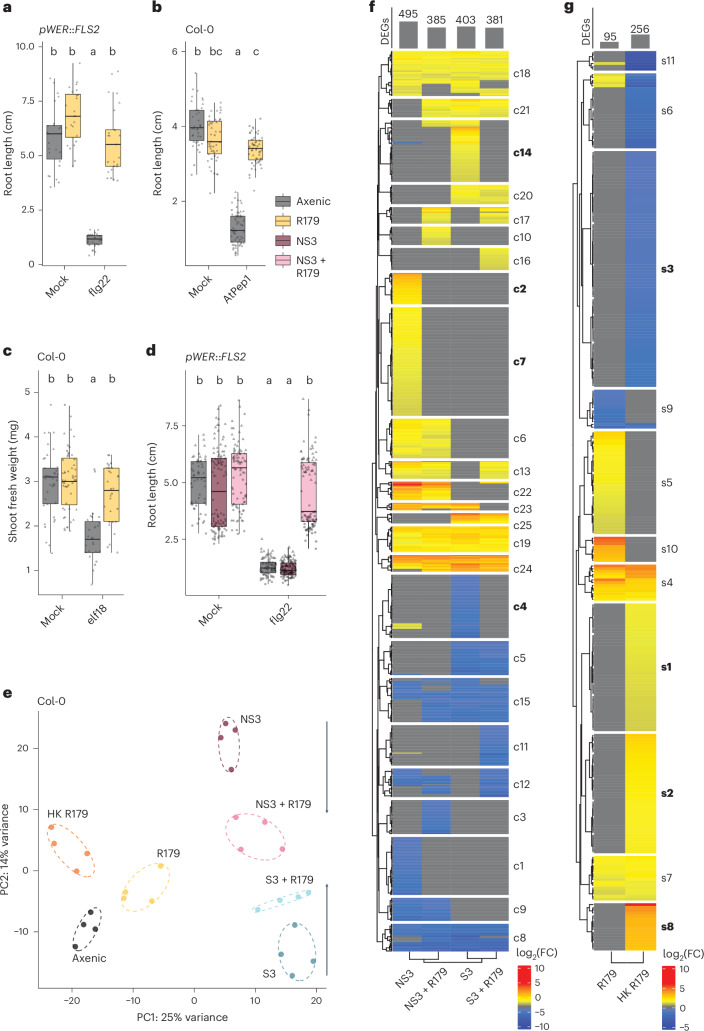
*Rhodanobacter* R179 suppresses plant growth inhibition induced by various MAMPs/DAMPs and modulates SynCom-induced root transcriptional responses. **a**–**c**, Root length (**a**,**b**) and shoot weight (**c**) of *Arabidopsis pWER*::*FLS2–GFP* (**a**) and wild-type Col-0 (**b**,**c**) co-inoculated for 2 weeks with 1 µM flg22 (**a**), 1 µM AtPep1 (**b**) or 1 µM elf18 (**c**) together with *Rhodanobacter* R179 (yellow; start inocula OD_600_ = 0.0005). **d**, Root length of *Arabidopsis pWER*::*FLS2–GFP* co-inoculated with 1 µM flg22 and a five-member non-immunosuppressive SynCom alone (dark purple), or in combination with R179 (light purple). The shapes represent replicates (3), with totals of *n* = 26, 24, 29 and 25 for **a**; *n* = 38, 46, 63 and 55 for **b**; *n* = 39, 52, 28 and 29 for **c**; and *n* = 57, 104, 89, 83, 104 and 101 for **d** measured primary roots/shoots of individual plants (from left to right within each panel). The letters indicate statistical significance determined using a two-sided Dunn’s test (*P* < 0.05; adjusted by Bonferroni correction of *P*). In the box plots in **a**–**d**, the horizontal line indicates the median, the boxes extend to the 25th and 75th percentiles and the whiskers extend to the furthest point within 1.5× the interquartile range. **e**, Principal component (PC) analysis plot showing the dissimilarity of root transcriptomes after 2 weeks of co-cultivation with the indicated bacteria or SynComs (start inoculum for each live bacterium OD_600_ = 0.0005). S3, immunosuppressive SynCom 3; NS3, non-immunosuppressive SynCom 3; HK R179, heat-killed R179 (OD_600_ = 1.5). The ellipses correspond to *t*-distributions fitted to each cluster (70% confidence interval). RNA sequencing was performed with samples from four independent replicates, each from 10–15 pooled roots (*n* = 4 per condition). The upward and downward arrows illustrate the convergence of transcriptomes by R179 co-inoculation. **f**, Heat map of DEGs in *Arabidopsis* roots after 2 weeks of colonization with the indicated SynComs (start inoculum for each live bacterium OD_600_ = 0.0005; log_2_(fold change) ≥ 1; *P* ≤ 0.05). *k*-means clusters (*k* = 25) are marked on the right. Total numbers of DEGs are indicated at the top. FC, fold change. **g**, Heat map of DEGs in roots after colonization with R179 in mono-association (start inocula OD_600_ = 0.0005) or exposure to HK R179 (OD_600_ = 1.5). *k*-means clusters (*k* = 11) are marked on the right. Total numbers of DEGs are indicated at the top. Source data

**Extended Data Fig. 2:**
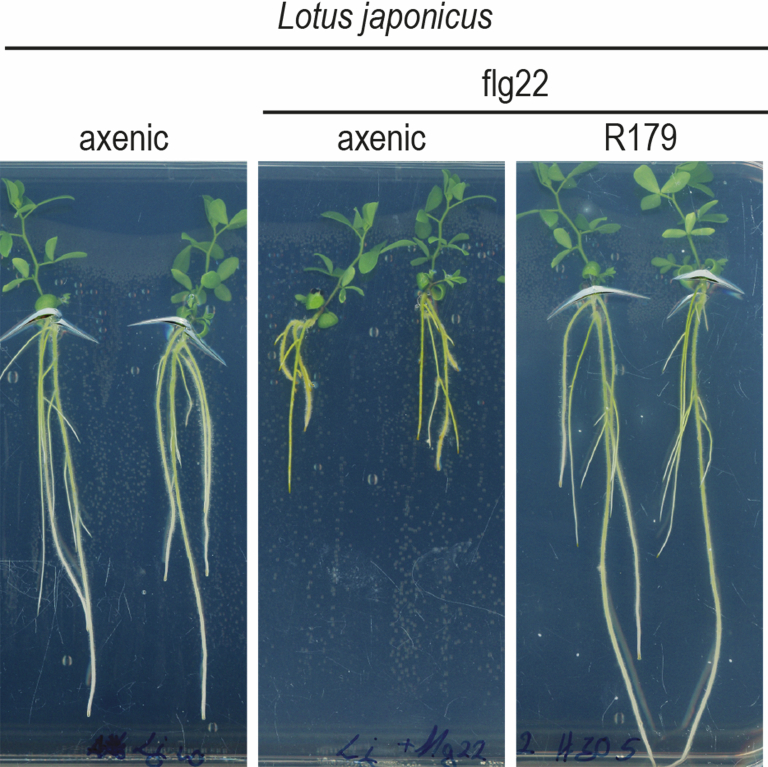
Suppression of flg22-induced root growth inhibition on *L. japonicus.* Phenotypes of three-week-old *L. japonicus* plants cultivated with *Rhodanobacter* R179 in presence or absence of 1 µM flg22 (start inocula OD_600_ = 0.0005).

We tested a subset of pathogenic or commensal Xanthomonadales strains derived from plant or algal hosts for suppression of RGI induced by the DAMP AtPep1 (Figs. 1a and 2b). All seven tested strains suppressed AtPep1-induced RGI, showing that the suppressive activity is not restricted to the flg22 elicitor. Treatment of *L. japonicus* with flg22 is known to elicit early immune responses such as reactive oxygen species spiking and transcriptional reprogramming^66,67^. We observed reduced root growth in *L. japonicus* accession Gifu upon chronic exposure to flg22. All seven strains tested for suppression of AtPep1-induced RGI also alleviated flg22-induced *L. japonicus* RGI (Fig. 1d and Extended Data Fig. 2). This indicates that immunosuppressive strategies employed by Xanthomonadales are effective on at least two plant species.

### Immunosuppressive *Rhodanobacter* modulates root transcription

We selected one suppressive Xanthomonadales strain, the *Arabidopsis* root commensal *Rhodanobacter* R179, which belongs to the deepest branch of the Xanthomonadales, for further in-depth analysis. As PTI-associated RGI was suppressed by leaf-derived *Xanthomonas* species in roots (Figs. 1a and 2a), we tested whether, vice versa, the alleviation of growth inhibition by root-derived R179 manifests in shoots (Fig. 2c). Shoot growth inhibition caused by the bacterial MAMP elf18, known to be predominantly recognized in leaves of *Arabidopsis*^68^, was suppressed by R179 (Fig. 2c). Thus, interference with PTI-associated growth restriction by R179 is conserved in roots and leaves for the MAMPs flg22 and elf18, as well as the DAMP AtPep1.

Co-inoculation of *Arabidopsis* with the five-member NS3 SynCom together with R179 resulted in the suppression of flg22-induced RGI (Fig. 2d). This prompted us to investigate whether R179 influences host defence-associated transcriptional responses during plant–microbiota interactions in the absence of exogenously applied immunogenic peptides. We assessed the root transcriptome of *Arabidopsis* Col-0 colonized for 2 weeks by R179 alone, or together with either a five-member immunosuppressive SynCom (S3 SynCom) or the NS3 SynCom, designed on the basis of the members’ ability to alleviate flg22-induced RGI in mono-associations (Supplementary Table 1). Variation in root transcriptomes was predominantly explained by SynCom colonization regardless of immunomodulatory activities—that is, root transcriptomes of plants colonized by the NS3 or S3 SynCom are separated from axenic plants or those colonized by R179 alone (first principal component, 25% of the variation explained; Fig. 2e). The second component distinguished transcriptional responses to the S3 SynCom and NS3 SynCom (14%; Fig. 2e). Interestingly, co-inoculations of the S3 or NS3 SynCom with R179 resulted in an overall convergence of the corresponding root transcriptomes—that is, R179 reduced the dissimilarity of transcriptional changes induced by either SynCom alone (arrows in Fig. 2e).

*k*-means hierarchical clustering of differentially expressed genes (DEGs) obtained by comparing treated roots with axenic roots identified clusters c2 and c7 as specifically induced by the NS3 SynCom. Consistent with an immunomodulatory role of R179, all DEGs in both clusters were absent upon R179 co-inoculation (Fig. 2f). Gene Ontology (GO) enrichment analyses revealed that clusters c2 and c7 are enriched in genes with functions related to regulation of defence response, defence response to fungus, response to salicylic acid and cell-surface receptor signalling (Supplementary Table 3). Upon colonization with the S3 SynCom, we observed downregulation of another partially defence-related cluster, c4, and upregulation of cluster c14, which is enriched in genes with functions related to regulation of phenylpropanoid metabolism and detection of bacteria (Supplementary Table 3). Differential transcriptional regulation of genes in these two clusters was also attenuated by R179 co-colonization. Taken together, the impact of R179 on the expression of SynCom-regulated clusters c2, c7, c4 and c14 explains how the commensal reduces the dissimilarity of root transcriptomes. Comparative transcriptomic analysis indicated that these clusters overlapped significantly with those identified in a previous study using the identical NS3 and S3 SynComs^44^. Genes shared between both studies show similar expression patterns and are enriched with functions related to defence, response to hypoxia, response to inorganic substance and cell wall modification (Supplementary Table 4). These results suggest that R179 root colonization suppresses defence responses triggered by either exogenously applied immunogenic peptides or live bacterial communities.

**Extended Data Fig. 3:**
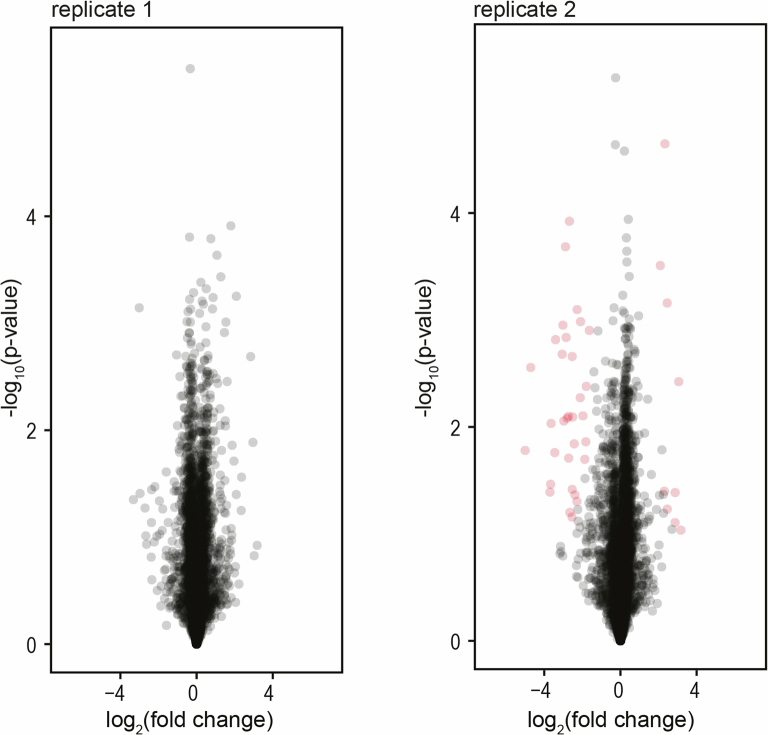
Root proteomics after R179 colonization. Comparative proteomics of axenic and *Rhodanobacter* R179 colonized roots from two-week-old *Arabidopsis* Col-0 plants. Red indicates proteins with significant changes between the axenic and colonized roots (t-test: *p* < 0.05; adjusted by Bonferroni correction of *P*). n = 3 biological replicates of pooled roots per condition and independent experiment. Source data

**Extended Data Fig. 4:**
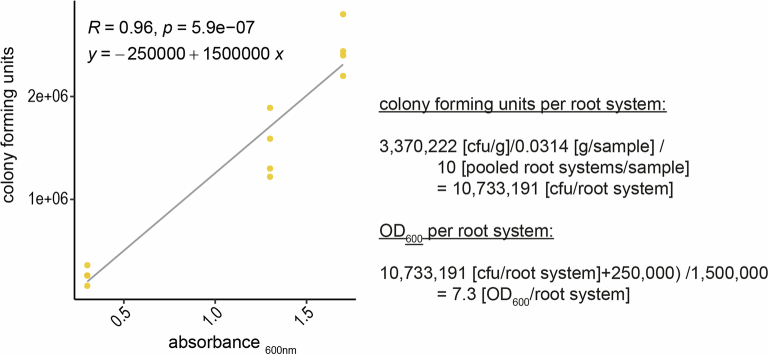
Linear regression of colony forming units and absorbance. Number of colony forming units of *Rhodanobacter* R179 in a dilution series and calculation of a root-specific OD_600_ based on average colonization data (in colony forming units) of root samples. n = 3 technical replicates of agar-plated bacterial suspension per culture of given absorbance. Source data

In contrast to prolonged co-cultivation of roots with SynComs for 2 weeks (381–495 DEGs), prolonged co-cultivation with R179 alone resulted in only minor transcriptional changes compared with axenic roots (95 DEGs; Fig. 2e,g). This was corroborated by proteomic analysis in which the relative abundance of none of the approximately 7,000 root proteins detected was significantly enriched or depleted upon R179 colonization (Extended Data Fig. 3). To investigate whether the apparent lack of root responses to live R179 colonization results from the evasion of host immune perception, we applied either live bacteria or an excess of heat-killed R179 (3,000-fold; optical density at 600 nm (OD_600_) = 1.5) to the plant growth medium (Fig. 2g). On the basis of a linear regression correlating OD_600_ and colony-forming units (CFU) (Extended Data Fig. 4) and the assumption that the activity of heat-killed bacteria will be reduced after 14 days of incubation, we expected the excess of heat-killed bacteria to be considerably less than the final titre of live R179 bacteria. Treatment with heat-killed R179 resulted in marked transcriptional responses exceeding responses to live R179 alone (256 DEGs) that are primarily attributable to four gene clusters, s1, s2, s3 and s8 (Fig. 2g). Cluster s8 was enriched in genes involved in cell-surface receptor signalling, pointing to the presence of heat-stable MAMPs in heat-killed R179 whose immunogenic activity is suppressed by live R179 during plant colonization (Supplementary Table 3).

### Immunomodulatory determinants enhance R179 competitiveness

**Fig. 3:**
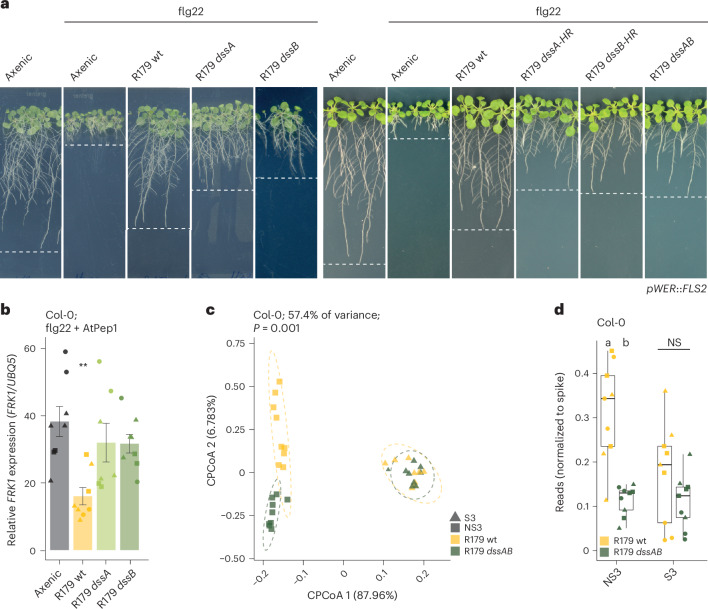
Immunosuppressive determinants *dssA* and *dssB* provide *Rhodanobacter* R179 with a competitive advantage in a community context. **a**, Phenotypes of 3-week-old *Arabidopsis pWER*::*FLS2–GFP* plants cultivated with *Rhodanobacter* R179 *dssA* and *dssB* mini-Tn5 insertion mutants, independent in-frame deletion mutants (*dssA-HR* and *dssB-HR*) and a *dssAB* double mutant plus 1 µM flg22 (start inocula OD_600_ = 0.0005). The dashed horizontal lines correspond to the approximate primary root length of plants under different treatments. wt, wild type. **b**, Expression of the defence marker gene *FRK1* normalized to *UBQ5* in roots of *pWER*::*FLS2–GFP* plants. The plants were pre-colonized for 14 days with *Rhodanobacter* R179 derivatives (start inocula OD_600_ = 0.0005) and flood-inoculated for 1 h with 1 µM AtPep1 and 1 µM flg22 followed by 4 h of rest. The bars represent means ± s.e.m. The asterisks indicate a statistically significant difference compared with axenic roots determined using a two-sided Dunn’s test (**P = 0.0026, adjusted by Bonferroni correction of *P*). The shapes indicate three replicates with a total of *n* = 8, 8, 7 and 8 biological replicates per condition consisting of pooled roots (from left to right). **c**, Constrained principal component analysis (CPCoA) of the normalized reads from the non-immunosuppressive (NS3, squares) and immunosuppressive (S3, triangles) SynComs on Col-0 roots after 2 weeks of colonization upon in silico depletion of R179-specific reads (start inoculum of each strain OD_600_ = 0.0005). The data correspond to three replicates with a total of *n* = 12 biological replicates per condition consisting of pooled roots. The ellipses correspond to Gaussian distributions fitted to each cluster (95% confidence interval). **d**, Spike-normalized abundance of wild-type *Rhodanobacter* R179 and the *dssAB* mutant on *Arabidopsis* roots upon co-cultivation with the non-immunosuppressive (NS3) or suppressive (S3) SynCom for 2 weeks. The letters indicate statistical significance determined using a two-sided Dunn’s test (*P* < 0.05; adjusted by Bonferroni correction of *P*; NS, no statistical difference). In the box plots, the horizontal line indicates the median, the boxes extend to the 25th and 75th percentiles and the whiskers extend to the furthest point within 1.5× the interquartile range. Source data

**Extended Data Fig. 5:**
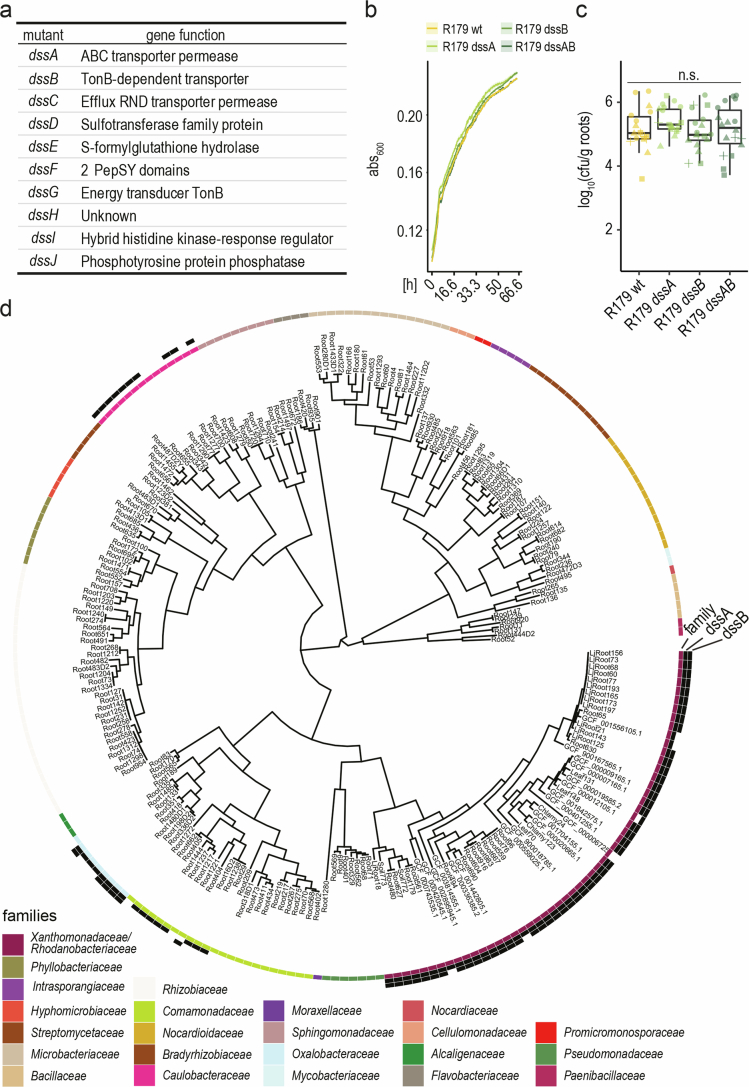
Impaired immunomodulation by *Rhodanobacter* R179 *dssA* and *dssB* mutants and occurrence of *dssA* and *dssB* orthologs in the *At*-R-SPHERE culture collection and Xanthomonadales. **a**, Functional annotation of transposon-disrupted genes of defence suppression system (*dss*) candidate mutants. **b**, Growth curves of wild-type *Rhodanobacter* R179 and *dss* mutants in XVM2 minimal medium for 66 h. Representative experiment of three replicates with n = 6 per bacterial strain. **c**, Bacterial titer represented as colony forming units (cfus) of wild-type *Rhodanobacter* R179 (yellow), the *dssA* (light green), and *dssB* (green) single mutants, and the *dssAB* double mutant (dark green) on wild-type *Arabidopsis* after two weeks of co-cultivation in mono-associations (start inocula OD_600_ = 0.0005). Shapes indicate four replicates with in total n = 18, 21, 18 and 18 biological replicates per condition of pooled roots (from left to right). Letters indicate statistically significant differences determined using two-sided Dunn’s test (*p* < 0.05; adjusted by Bonferroni correction of *P*, n.s: no statistical difference). The box plots center on the median and extend to the 25th and 75th percentiles, and the whiskers extend to the furthest point within 1.5× the interquartile range. **d**, Occurrence of *dssA* and *dssB* orthogroups across different bacterial families indicated as the second and outermost ring. Source data

We used long-read sequencing to assemble and annotate a finished R179 genome. This assembly lacks operons for known virulence factors of bacterial phytopathogens, such as the T3SS machinery^69^, biosynthesis genes of the phytohormone mimic coronatine^70^, the flg22-degrading protease *aprA*^71^ and the PQQ biosynthesis operon (required for gluconic acid production)^42^. We conducted a mutant screen using *Rhodanobacter* R179 in planta to identify bacterial determinants contributing to the immunomodulatory activity. We generated 7,100 *mini-Tn5* (ref. ^72^) insertion mutants in the R179 background and tested them individually for their ability to suppress flg22-induced RGI in the *Arabidopsis pWER*::*FLS2* line using a high-throughput phytostrips-based assay^73^. A total of 67 candidates that showed partial loss of suppressive activity in two independent biological replicates were re-evaluated for impaired suppressive activity in *Arabidopsis* using conventional square agar plates (Fig. 3a). We identified ten *dss* mutant candidates with varying levels of reduced suppression of flg22-induced RGI (Fig. 3a and Extended Data Fig. 5a) and further characterized two of them.

Pre-colonization with wild-type R179, but neither the *dssA* nor the *dssB* mutant, inhibited induced expression of the defence marker gene *FRK1* in *Arabidopsis* Col-0 roots 5 h after co-application of flg22 and AtPep1 peptides (Fig. 3b). The R179 *dssA* and *dssB* mutants have *mini-Tn5* transposon insertions in the *ABC transporter permease* and *TonB-dependent transporter* genes, respectively (Extended Data Fig. 5a and Supplementary Table 5). The *ABC transporter permease dssA* is encoded within an operon encompassing 17 genes, whereas the *TonB-dependent transporter dssB* is predicted to be transcribed as monocistronic mRNA. Independent in-frame deletion mutants of *dssA* and *dssB* generated by homologous recombination exhibited impaired suppression of RGI induced by flg22, similar to the corresponding *mini-Tn5* transposon insertion mutants, suggesting that the partial loss of RGI suppression is due to the disruption of the *dssA* and *dssB* genes rather than potential off-target effects (Fig. 3a). We generated a double mutant (*dssAB*) via in-frame deletion of *dssA* through homologous recombination in the *dssB mini-Tn5* insertion mutant background. The resulting R179 *dssAB* double mutant also failed to suppress flg22-induced RGI (Fig. 3a). The in-frame deletions and the *mini-Tn5* insertions were verified by long-read PacBio sequencing, and no additional chromosomal rearrangements were detected. The R179 *dssA* and *dssB* single mutants and the *dssAB* double mutant showed wild-type-like growth in liquid XVM2 minimal medium as well as wild-type-like colonization of *Arabidopsis* roots when inoculated in mono-associations (Extended Data Fig. 5b,c).

**Extended Data Fig. 6:**
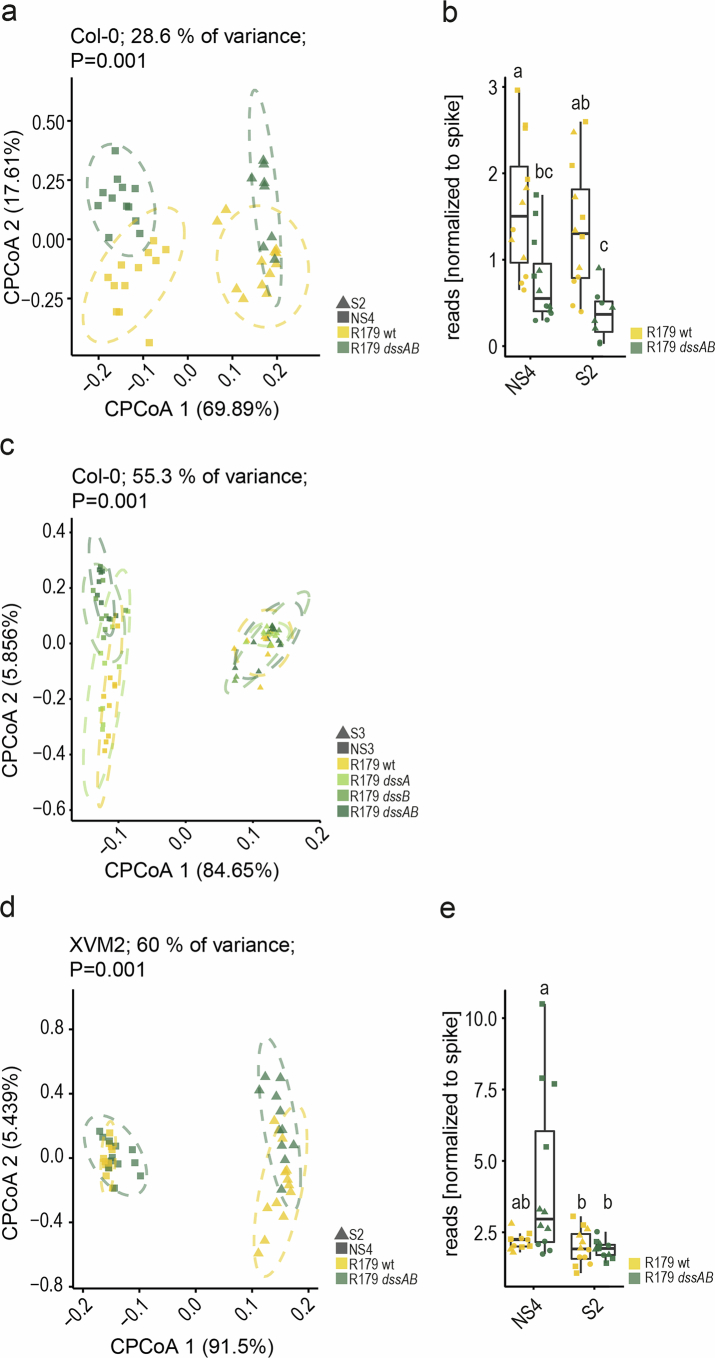
Immunosuppressive determinants *dssA* and *dssB* of *Rhodanobacter* R179 shape the composition of a bacterial community on roots in absence of other immunosuppressive bacteria. **a**, Constrained coordination of the normalized reads from the non-immunosuppressive (NS4, squares) and immunosuppressive (S2, triangles) SynCom on Col-0 roots after two-weeks colonization upon *in silico* depletion of R179-specific reads (start inoculum of each strain OD_600_ = 0.0005). Data correspond to three replicates with in total n = 12 biological replicates per condition consisting of pooled roots. Ellipses correspond to Gaussian distributions fitted to each cluster (95% confidence interval). **b**, Spike-normalized abundance of wild-type *Rhodanobacter* R179 and the *dssAB* mutant of samples described in a. Letters indicate statistical significance determined using two-sided Dunn’s test (*p* < 0.05; adjusted by Bonferroni correction of *P*; n.s: no statistical difference). The box plots of panel b and e both center on the median and extend to the 25th and 75th percentiles, and the whiskers extend to the furthest point within 1.5× the interquartile range. **c**, Constrained coordination of the normalized reads from the non-immunosuppressive (NS3, squares) and immunosuppressive (S3, triangles) SynCom on Col-0 roots upon co-colonization with indicated R179 variants. Data correspond to three replicates with in total n = 9 biological replicates per condition consisting of pooled roots. Ellipses correspond to Gaussian distributions fitted to each cluster (95% confidence interval). A subset of the data has been shown in Fig. 3c. **d**, Constrained coordination of the normalized reads from the non-immunosuppressive (NS4, squares) and immunosuppressive (S2, triangles) SynCom upon cultivation in liquid XVM2 medium. R179-specific reads were depleted *in silico*. Data correspond to three replicates with in total n = 12 biological replicates per condition consisting of pooled roots. Ellipses correspond to Gaussian distributions fitted to each cluster (95% confidence interval). **e**, Spike-normalized abundance of wild-type *Rhodanobacter* R179 and the *dssAB* mutant of samples described in d. Letters indicate statistical significance determined using two-sided Dunn’s test (*p* < 0.05; adjusted by Bonferroni correction of *P*; n.s: no statistical difference). The box plots centre on the median and extend to the 25th and 75th percentiles, and the whiskers extend to the furthest point within 1.5× the interquartile range. Source data

We next tested whether mutations of R179 *dssA* or *dssB* or combined mutations of both genes alter the establishment of synthetic microbiota on *Arabidopsis* Col-0 roots. Wild-type R179 and mutants were tagged with a recently developed MoBacTag barcode, facilitating strain-specific tracking during community profiling in planta^74^. Upon co-inoculation of *Arabidopsis* with four phylogenetically matching five-member suppressive (S2 and S3) or non-suppressive SynComs (NS3 and NS4) with either wild-type R179 or the *dssAB* mutant, we conducted a constrained principal component analysis of Bray–Curtis dissimilarities on the SynCom-member-specific 16S rRNA reads normalized to spike reads (Fig. 3c, Extended Data Fig. 6a and Supplementary Table 1). This revealed that wild-type R179 and its *dssAB* double mutant differentially influenced the composition of both non-suppressive SynComs, whereas the compositions of the suppressive SynComs were indistinguishable regardless of co-colonization with wild-type R179 or the *dssAB* mutant. Co-colonization of R179 *dssA* or *dssB* single mutants resulted in an intermediate NS3 composition compared with the composition in the presence of wild-type R179 or the *dssAB* double mutant (Extended Data Fig. 6c). The inactivation of *dssA* and *dssB* thus appears to perturb the establishment of the community more strongly than that of the individual mutants.

Spike-normalized abundance of wild-type R179 was significantly elevated compared with the *dssAB* mutant on roots when inoculated together with both non-suppressive SynComs (NS3 and NS4) and the S2 suppressive SynCom (Fig. 3d and Extended Data Fig. 6b). By contrast, in liquid medium without plants, the abundances of wild-type R179 and the *dssAB* double mutant were comparable for both SynComs (Extended Data Fig. 6e). Furthermore, in liquid cultures, a similar composition of the non-suppressive SynCom (NS4) was observed regardless of co-cultivation with wild-type R179 or the *dssAB* double mutant, and the composition of the suppressive SynCom (S2) shifted only slightly when wild-type R179 or the *dssAB* mutant were co-cultivated (Extended Data Fig. 6d). This suggests that wild-type alleles of *dssA* and *dssB* provide R179 with a competitive advantage specifically in root-associated bacterial communities.

Orthogroup prediction using genomes of the *At*-R-SPHERE commensal culture collection and Xanthomonadales isolated from diverse environments showed that *dssA* and *dssB* are highly conserved within the Xanthomonadales and typically lacking in other phylogenetic lineages of the *Arabidopsis* bacterial root microbiota (Extended Data Fig. 5d). Within the Xanthomonadales, only a representative genome of the specialized pathogen *Xylella fastidiosa*, which has a significantly smaller genome than the average *Xanthomonas* species^75^, lacked orthologues of *dssA* and *dssB*. Outside the Xanthomonadales clade within the *At*-R-SPHERE culture collection^5^, only some Oxalobacteriaceae harbour genes belonging to both *dssA* and *dssB* orthogroups. A BLAST-based search for *dssA* and *dssB* orthologous genes in publicly available Xanthomonadales draft genomes revealed that 74.7% and 88.9% of Xanthomonadales genomes harbour *dssA* and *dssB* orthologues, respectively (Supplementary Tables 10 and 11). Evolutionary conservation of *dssA* and *dssB* orthologues in Xanthomonadales thus correlates with the conserved capability of Xanthomonadales to suppress PTI-associated RGI (Fig. 1a and Extended Data Fig. 5d).

### *Rhodanobacter* R179 eliminates immunogenic peptides

**Extended Data Fig. 7:**
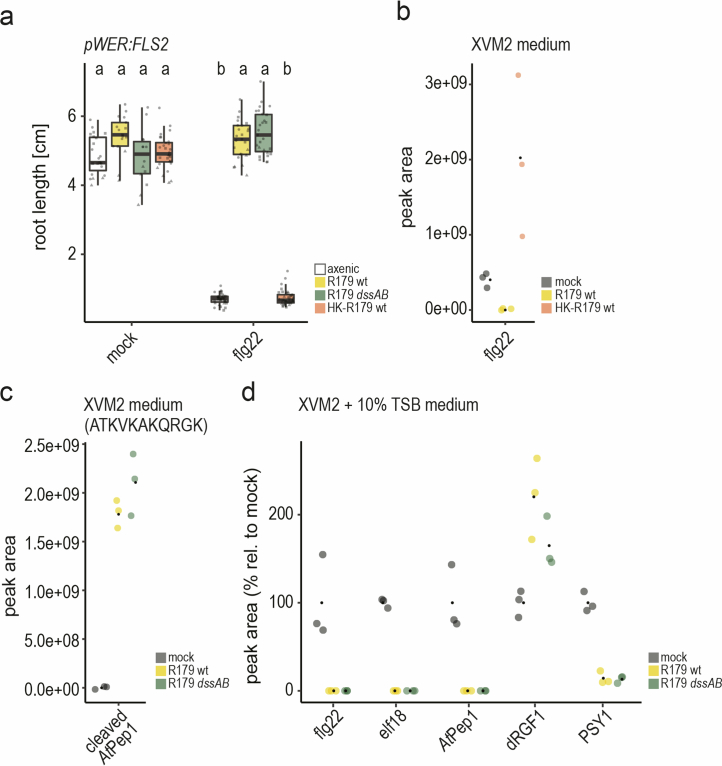
Elimination of immunogenic epitopes through cell-free supernatants of *Rhodanobacter* R179. **a**, Root length of *Arabidopsis pWER:FLS2-GFP* incubated with 1 µM flg22 and filter-sterile supernatant of wild-type *Rhodanobacter* R179 (yellow), the *dssAB* mutant (green) or heat-killed (HK) wild-type R179 (red) supernatant for two-weeks (bacterial inoculum used: OD_600_ = 0.4). Shapes indicate three replicates with in total n = 23, 17, 14, 28, 32, 25, 27 and 37 measured primary roots of individual plants (from left to right). Letters indicate statistical significance determined using two-sided Dunn’s test (*p* < 0.05; adjusted by Bonferroni correction of *P*). The box plots center on the median and extend to the 25th and 75th percentiles, and the whiskers extend to the furthest point within 1.5× the interquartile range. **b**, Quantification of flg22 by mass spectrometry after 1 h incubation with filter-sterile supernatant (yellow) and heat-treated supernatant (red) of wild-type R179 cultivated in XVM2 medium (bacterial inoculum used OD_600_ = 0.4). Black points represent the mean. n = 3 culture supernatants of individual bacterial colonies. **c**, Quantification of an identified AtPep1 cleavage product by mass spectrometry after 1 h incubation with filter-sterile supernatant of wild-type R179 (yellow) or *dssAB* mutant cultivated in XVM2 medium (bacterial inoculum used OD_600_ = 0.4). Black points represent the mean. n = 3 culture supernatants of individual bacterial colonies. **d**, Quantification of indicated peptides after incubation with supernatants of bacteria cultivated in XVM2 minimal medium supplemented with 10% TSB. n = 3 culture supernatants of individual bacterial colonies. A subset of the data has been shown in Fig. 4. Source data

Since the *dssAB* double mutant exhibits residual suppression of flg22-induced RGI (Fig. 3a), we hypothesized that R179 has additional means to interfere with PTI. We pre-cultured R179 in XVM2, a minimal medium, which is known to induce virulence gene expression in phytopathogenic *Xcv*^76^, and tested the bacteria-free R179 supernatant for the ability to suppress flg22-induced RGI in *Arabidopsis pWER*::*FLS2*. The supernatant of wild-type R179 contained immunosuppressive activities that were retained in the supernatant of the *dssAB* mutant (Extended Data Fig. 7a). Heat treatment of the supernatant abolished the immunosuppressive activity, suggesting the involvement of a secreted, soluble and heat-labile factor (Extended Data Fig. 7a). As suppression of flg22-induced RGI was also observed for the supernatant of the R179 *dssAB* mutant, R179 probably interferes with PTI via at least two distinct mechanisms.

**Fig. 4:**
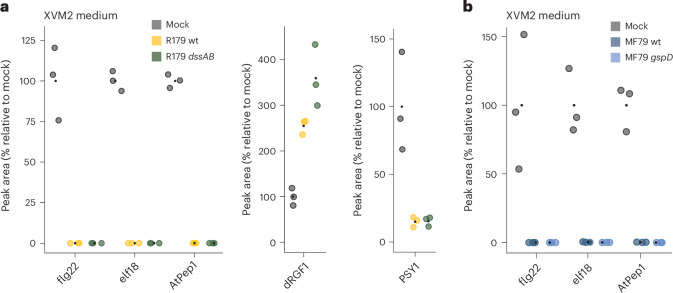
Selective peptide elimination by the cell-free supernatant of Rhodanobacteraceae*.* **a**, Peptide quantification by mass spectrometry after 1 h of incubation in XVM2 minimal medium or the filter-sterile supernatant of wild-type *Rhodanobacter* R179 (yellow) or the *dssAB* mutant (green) cultivated in XVM2 medium (bacterial culture: OD_600_ = 0.4). The black points represent the mean. *n* = 3 culture supernatants of individual bacterial colonies. **b**, Peptide quantification by mass spectrometry after 1 h of incubation in XVM2 minimal medium or the filter-sterile supernatant of wild-type *D. japonica* MF79 (dark blue) or the *gspD* mutant (light blue) cultivated in XVM2 medium (bacterial culture: OD_600_ = 0.6). Source data

We hypothesized that the observed immunosuppressive activity of the R179 supernatant may be caused by secreted peptidase(s) that are often sensitive to heat treatment. We quantified flg22 via mass spectrometry after 1 h of incubation in supernatant collected from wild-type R179 or the *dssAB* mutant or heat-treated supernatant of wild-type R179. We detected flg22 peptide after incubation in the heat-treated supernatant but not the supernatant of wild-type R179 or the *dssAB* mutant, indicating efficient and rapid (1 h) proteolytic degradation of the flg22 peptide in vitro (Fig. 4a and Extended Data Fig. 7b). Of note, two other immunogenic peptides, N-acetylated elf18 and AtPep1 were depleted in the supernatants of both wild-type R179 and the R179 *dssAB* mutant, whereas the root-growth-promoting sulfated PSY1 peptide and the de-sulfated dRGF1 peptide were still detected (Fig. 4a)^77,78^. Unlike flg22 and elf18, minute traces of AtPep1 (<0.01%) were detected, and its reduced abundance correlated with the detection of a new peak corresponding to an AtPep1 cleavage product (ATKVKAKQRGK), indicating that AtPep1 reduction is probably caused by proteolytic cleavage (Extended Data Fig. 7c). To exclude possible starvation-induced expression of peptidases upon cultivation in minimal medium, we repeated the experiment using supernatant of R179 grown in XVM2 medium supplemented with 10% carbon-rich medium (tryptic soy broth) and obtained similar results (Extended Data Fig. 7d). We concluded that the R179 secretome suppresses PTI through selective elimination of MAMPs/DAMPs via mechanisms independent of *dssA* and *dssB*.

The culture supernatant from root commensal *D. japonica* MF79, a relative of *Rhodanobacter* R179, was similarly effective in degrading flg22, elf18 and AtPep1 after 1 h of incubation (Fig. 4b). This indicates that the secretion of peptidase(s) eliminating immunogenic peptides is a common mechanism employed by these two Xanthomonadales strains. The supernatant of the *D. japonica* MF79 *gspD* mutant also depleted the immunogenic peptides flg22, AtPep1 and elf18. Hence, an intact *D. japonica* MF79 T2SS is dispensable for the elimination of the immunogenic peptides tested (Fig. 4b).

### Strain-specific factors determine Xanthomonadales root load

**Fig. 5:**
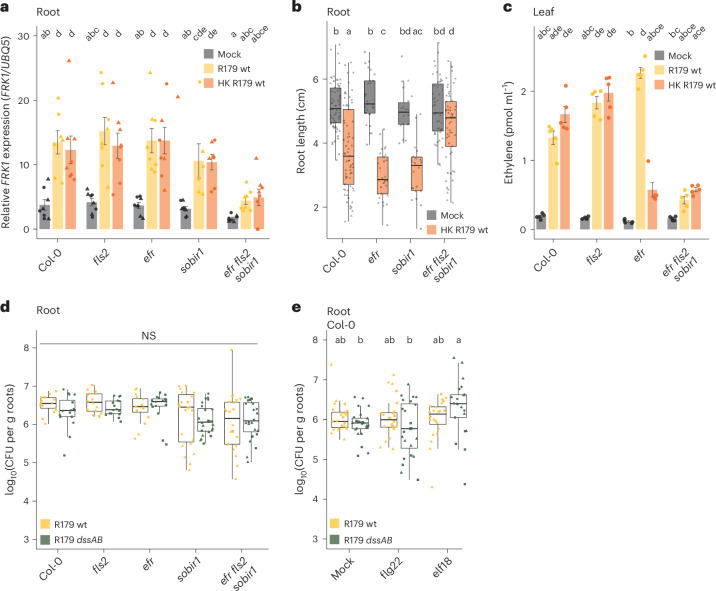
PRR-dependent recognition of *Rhodanobacter* R179 does not affect the colonization of *Arabidopsis* in mono-association. **a**, Expression of *FRK1* normalized to *UBQ5* in roots of wild-type and PRR mutant plants 5 h after application of live (yellow) or HK R179 (red, OD_600_ = 1.5). The bars represent means ± s.e.m. The letters indicate statistically significant differences determined using a two-sided Dunn’s test (*P* < 0.05; adjusted by Bonferroni correction of *P*). The shapes indicate two replicates with a total of *n* = 8, 7, 8, 8, 8, 8, 8, 8, 8, 8, 6, 7, 8, 8 and 8 biological replicates per condition consisting of pooled roots (from left to right). **b**, Root length of wild-type *Arabidopsis* and PRR mutant plants incubated with HK R179 (red, OD_600_ = 1.5) for 2 weeks. The letters indicate statistically significant differences determined using a two-sided Dunn’s test (*P* < 0.05; adjusted by Bonferroni correction of *P*). The shapes indicate two to three replicates with a total of *n* = 68, 69, 29, 29, 18, 22, 47 and 57 measured primary roots of individual plants (from left to right). In the box plots, the horizontal line indicates the median, the boxes extend to the 25th and 75th percentiles and the whiskers extend to the furthest point within 1.5× the interquartile range. **c**, Ethylene production in leaf pieces of wild-type and PRR mutant plants 5 h after application of native (yellow) or HK R179 lysates (red, 0.1% (w/v)). The bars represent means ± s.e.m. The letters indicate statistically significant differences determined using a two-sided Dunn’s test (*P* < 0.05; adjusted by Bonferroni correction of *P*). The experiment was performed once with *n* = 5 biological replicates per condition consisting of pooled leaf pieces. **d**,**e**, Bacterial titres of wild-type *Rhodanobacter* R179 (yellow) and the *dssAB* mutant (green) on wild-type *Arabidopsis* (**d**,**e**) and PRR mutants (**d**) after 2 weeks of co-cultivation in mono-associations (start inocula OD_600_ = 0.0005). In **e**, the plants were additionally treated with 1 µM flg22 and 1 µM *E. coli*-derived elf18. The letters indicate statistically significant differences determined using a two-sided Dunn’s test (*P* < 0.05; adjusted by Bonferroni correction of *P*). The shapes indicate three replicates with a total of *n* = 17, 16, 16, 16, 16, 16, 23, 24, 24 and 24 for **d** and *n* = 24, 24, 24, 24, 24 and 21 for **e** biological replicates per condition consisting of pooled roots (from left to right). In the box plots, the horizontal line indicates the median, the boxes extend to the 25th and 75th percentiles and the whiskers extend to the furthest point within 1.5× the interquartile range. Source data

**Extended Data Fig. 8:**
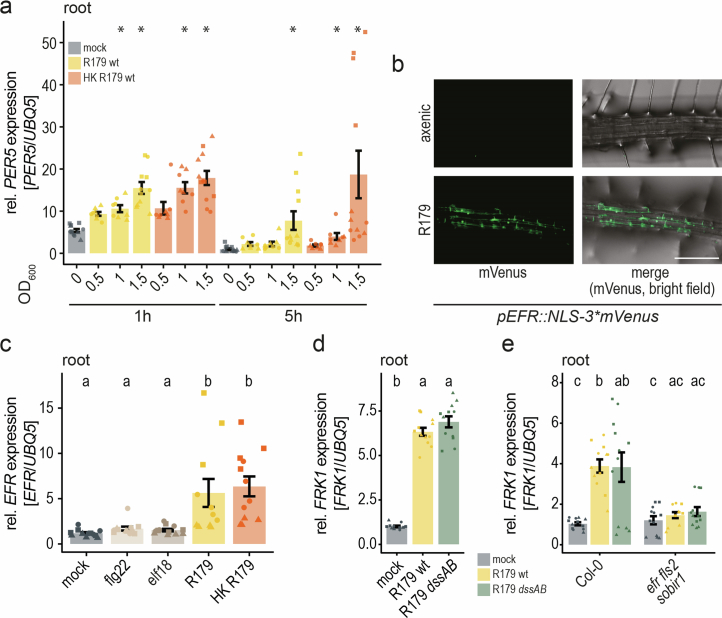
*Rhodanobacter* R179 induces the expression of *EFR* and *FRK1* in *Arabidopsis* roots. **a**, Expression of the defence marker gene *FRK1* normalized to *UBQ5* in roots of wild-type plants 1 and 5 h after application of a dilution series of live (yellow) or heat-killed (HK) R179 (red, OD_600_ = 0.5-1.5). Bars represent means ± s.e. Shapes indicate three replicates with in total n = 12, 8, 8, 11, 8, 8, 12, 12, 8, 8, 12, 8, 8 and 12 biological replicates consisting of pooled roots (from left to right). Asterisks indicate statistically significant differences compared to mock treatment for each timepoint determined using two-sided Dunn’s test (*: *p* < 0.05; adjusted by Bonferroni correction of *P*). **b**, Expression pattern of *EFR* in roots of the seven-day-old *Arabidopsis* seedlings using the reporter line pEFR:NLS-3*mVenus 20 h after treatment with *Rhodanobacter* R179 (OD_600_ = 1.5). Scale bar, 200 µm. **c**, Expression of *EFR* normalized to *UBQ5* in roots of *Arabidopsis* Col-0 5 h after application of live or heat-killed (HK) R179 (OD_600_ = 1.5), 1 µM flg22 or 1 µM elf18. Bars represent means ± s.e. Shapes indicate three replicates with in total n = 12, 12, 12, 11 and 12 biological replicates consisting of pooled roots (from left to right). Letters indicate statistically significant differences determined using two-sided Dunn’s test (*p* < 0.05; adjusted by Bonferroni correction of *P*). **d, e**, Expression of *FRK1* normalized to *UBQ5* in roots of *Arabidopsis* Col-0 5 h after application of bacteria (**e**) or the supernatant of bacteria (**d**) R179 wild-type (yellow) or the *dssAB* double mutant (green) pre-cultured in rich medium (0.5 TSB, harvested at OD_600_ = 1.5. Shapes indicate three replicates with in total n = 12 (for **d**) and n = 12, 12, 12, 12, 11 and 12 (from left to right for **e**) biological replicates consisting of pooled roots. Bars represent means ± s.e. Shapes indicate three replicates with in total 10 - 12 biological replicates consisting of pooled roots. Letters indicate statistically significant differences determined using two-sided Dunn’s test (*p* < 0.05; adjusted by Bonferroni correction of *P*). Source data

The transcriptomic analysis of *Arabidopsis* roots subjected to prolonged exposure to live and heat-killed R179 provided evidence that the commensal can both induce and suppress immune sectors in roots (Fig. 2g). The finding that R179 harbours immunogenic patterns was supported by the concentration-dependent induction of the expression of the *FRK1* defence marker gene in roots 5 h after treatment with live or heat-killed R179 (Fig. 5a and Extended Data Fig. 8a). The supernatant of the *Rhodanobacter* R179 *dssAB* mutant induced *FRK1* expression to a similar extent as the supernatant of wild-type R179, suggesting that the immunogenic activity of R179 functions independently of *dssA* and *dssB* (Extended Data Fig. 8d). We sought to identify *Arabidopsis* determinants required for R179 recognition using immune receptor kinase mutants. We found wild-type-like inducible *FRK1* root expression in plant mutants lacking the receptors FLS2 or EFR or the adaptor kinase SOBIR1 (Fig. 5a). Wild-type-like responsiveness of the *fls2* mutant is not surprising as the R179 genome lacks *fliC*, the source of the flg22 epitope. *FRK1* expression induced by wild-type R179 or the *dssAB* double mutant was essentially abolished in the roots of the *efr* *fls2* *sobir1* triple mutant (Fig. 5a and Extended Data Fig. 8e), suggesting redundant immune perception of the commensal by the EFR and SOBIR1 cell-surface receptor complexes, which is independent of *dssA* and *dssB*.

The colonization of wild-type *Arabidopsis* Col-0 with live R179 suppressed PTI-associated RGI and did not negatively affect root growth (Fig. 2a–d). By contrast, heat-killed R179 induced RGI, which was abolished in *efr* *fls2* *sobir1* plants (Fig. 5b), confirming that root growth restriction is a bona fide PRR-triggered and PTI-associated response to the commensal. Although *EFR* is generally believed to be expressed in shoots but not in roots^68^, we verified an R179-inducible root signal in an *EFR* transcriptional reporter line (pEFR::NLS–3*mVenus)^79,80^, which was further supported by the detection of endogenous *EFR* root transcripts upon R179 inoculation, but not elf18 application, in wild-type *Arabidopsis* (Extended Data Fig. 8b,c). Thus, R179-derived molecules other than elf18 seem to induce the expression of *EFR*, which subsequently contributes to R179 detection by the plant immune system in roots. Taken together, these data show redundant recognition of the R179 commensal by EFR and SOBIR1 cell-surface receptor complexes in roots.

**Extended Data Fig. 9:**
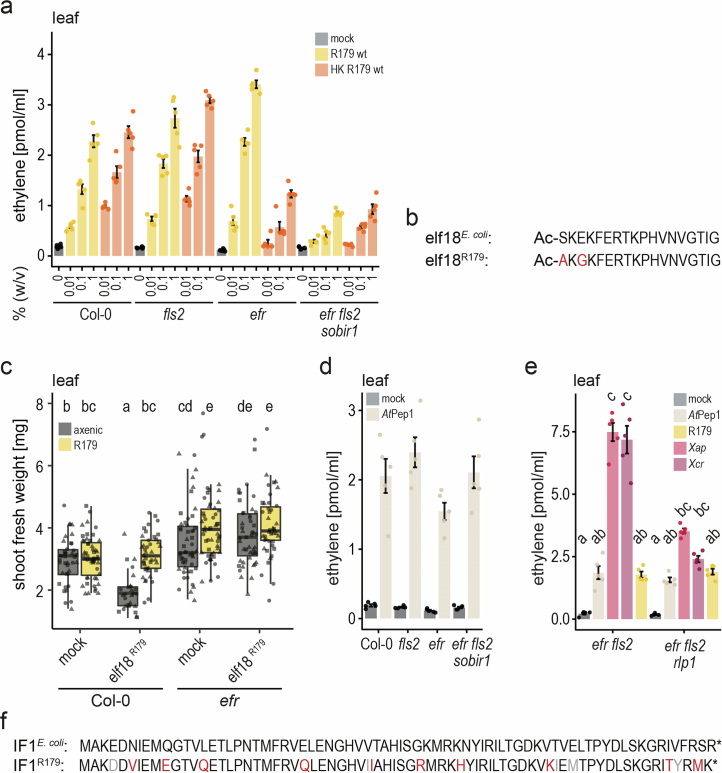
R179 recognition and immunosuppression in *Arabidopsis* leaves. **a**, Ethylene production in leaf pieces of wild type and PRR mutant plants 5 h after application of a dilution series of native (yellow) or heat-killed (HK) R179 lysates (red). A subset of the data has been shown in Fig. 5c. Bars represent means ± s.e. The experiment was performed once with n = 5 biological replicates per condition of pooled leave pieces. Letters indicate statistically significant differences determined using two-sided Dunn’s test (*p* < 0.05; adjusted by Bonferroni correction of *P*). **b**, Amino acid sequences of elf18^*E. coli*^ and elf18^R179^. Red color indicates amino acids with polymorphism. **c**, Shoot weight of *Arabidopsis* wild type and *efr* mutant plants incubated for two-weeks with 1 µM elf18 derived from R179 (elf18^R179^) together with *Rhodanobacter* R179 (yellow, start inocula OD_600_ = 0.0005). Shapes represent three replicates with in total n = 39, 52, 27, 41, 51, 52, 47 and 42 measured shoots of individual plants (from left to right). Letters indicate statistical significance determined using two-sided Dunn’s test (*p* < 0.05; adjusted by Bonferroni correction of *P*). The box plots center on the median and extend to the 25th and 75th percentiles, and the whiskers extend to the furthest point within 1.5× the interquartile range. A subset of the data has been shown in Fig. 2c. **d**, Ethylene production in leaf pieces of wild type and PRR mutant plants 5 h after application of 1 µM AtPep1. Bars represent means ± s.e. The experiment was performed once with n = 5 biological replicates per condition of pooled leave pieces. A subset of the data has been shown in Fig. 5c. **e**, Ethylene production in leaf pieces of wild type and PRR mutant plants 5 h after application of 1 µM AtPep1, or 0.1% (w/v) of the lysate of *Rhodanobacter* R179, *Xanthomonas arboricola* pv. *pruni* (*Xap*) and *Xanthomonas campestris pv. raphani* (*Xcr*). The experiment was performed once with n = 5 biological replicates per condition of pooled leave pieces. Bars represent means ± s.e. Letters indicate statistically significant differences determined using two-sided Dunn’s test (*p* < 0.05; adjusted by Bonferroni correction of *P*). **f**, Amino acid sequence of IF^*E. coli*^ and IF^R179^. Colors indicate amino acids with polymorphism. Source data

As R179 also alleviated shoot growth inhibition upon prolonged exposure to elf18 (Fig. 2c), we examined ethylene biosynthesis as another early plant immune response in leaves 5 h after treatment with native or heat-inactivated lysates of R179. Dose-dependent ethylene production was indistinguishable between wild-type *Arabidopsis* and the *fls2* mutant, but it was almost abolished in *efr* *fls2* *sobir1* (Fig. 5c and Extended Data Fig. 9a). Unexpectedly, ethylene production in the *efr* mutant was significantly reduced upon treatment with heat-inactivated but not native R179 (Fig. 5c). This suggests that a dominant heat-stable elicitor (or elicitors) of R179 is detected through EFR to induce ethylene production in leaves, which is masked by heat-labile MAMPs recognized in an EFR-independent but SOBIR1-dependent manner. This differs from the observed induction of defence marker gene expression in roots, where no heat-labile immunogenic activity was found with live R179, and where the response to heat-killed R179 was not affected in the *efr* single mutant. The genome of R179 encodes the cognate ligand of EFR, EF-Tu. We synthesized the corresponding peptide epitope elf18^R179^, differing from the commonly used elf18 elicitor by two amino acids, and verified EFR-dependent shoot growth inhibition induced by elf18^R179^ (Extended Data Fig. 9b,c). Moreover, R179 colonization suppressed shoot growth inhibition induced by exogenously administered elf18^R179^ or elf18^*E. coli*^ (Fig. 2c and Extended Data Fig. 9c). This supports the notion that camouflage by R179 in shoots involves the elimination of elf18^R179^ (Fig. 4a).

We observed ethylene production in *efr* *fls2* *sobir1* leaves following AtPep1 treatment that is indistinguishable from that in wild-type *Arabidopsis* (Extended Data Fig. 9d), indicating that the triple mutant can still mount PTI-associated defence upon PRR activation. We tested whether R179 produces the *Xanthomonas*-specific, heat-labile MAMP eMAX, whose perception is SOBIR-dependent, but we did not detect eMAX receptor (RLP1)-dependent ethylene production (Extended Data Fig. 9e)^81,82^. Instead, bacterial initiation factor 1, recently identified as a SOBIR1-dependent heat-labile bacterial MAMP, is encoded by R179 and might contribute to commensal perception (Extended Data Fig. 9f)^83^. Collectively, our data reveal the detection of at least three MAMPs of R179 by *Arabidopsis* (elf18^R179^, heat-labile and heat-stable MAMPs perceived by SOBIR1-dependent PRRs).

**Extended Data Fig. 10:**
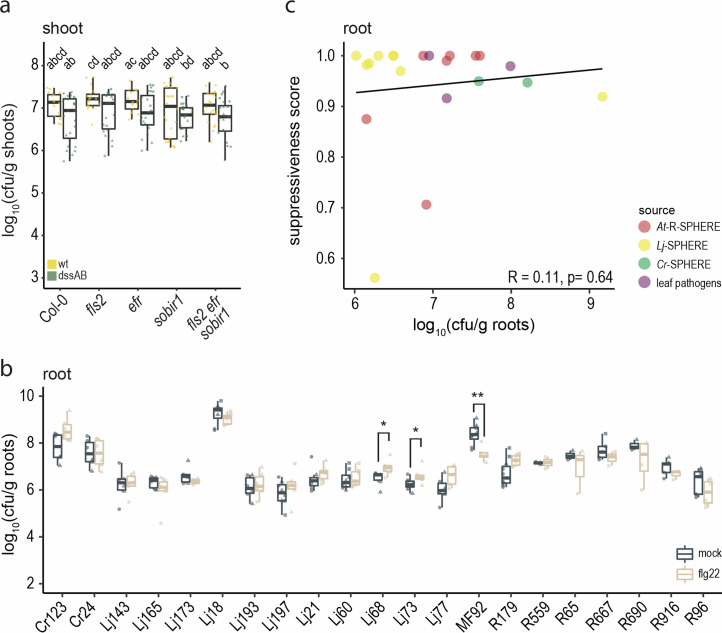
Proliferation of Xanthomonadales vary 1000-fold in planta. **a**, Bacterial titer represented as colony forming units (cfu) of wild-type *Rhodanobacter* R179 (yellow) and the *dssAB* mutant (green) on leaves of wild-type *Arabidopsis* and indicated PRR mutants after two weeks of co-cultivation in mono-associations (start inocula OD_600_ = 0.0005). Letters indicate statistically significant differences determined using two-sided Dunn’s test (*p* < 0.05; adjusted by Bonferroni correction of *P;* n.s: no statistical difference). Shapes indicate three replicates with in total n = 16, 24, 16, 22, 16, 24, 24, 24, 24 and 24 biological replicates per condition of pooled roots (from left to right). The box plots of panel a and b center on the median and extend to the 25th and 75th percentiles, and the whiskers extend to the furthest point within 1.5× the interquartile range. **b**, Quantification of indicated Xanthomonadales strains on roots of *Arabidopsis pWER:FLS2-GFP* in the presence or absence of 1 µM flg22. Asterisks indicate statistically significant differences determined using two-sided Wilcox test (*: *p* < 0.05, **: *p* < 0.01). Data correspond to biological samples used in Fig. 1a. **c**, Linear regression of colony forming units and suppressiveness of flg22-induced root growth inhibition. Average colonization of selected bacteria from indicated culture collections on mock- and flg22-treated *Arabidopsis pWER:FLS2-GFP* roots correlated with the suppressiveness score of the respective bacterium (suppressiveness score: difference in root length of flg22- to mock-treated plants in relation to the difference in root length of axenic flg22- vs. mock-treated plants). Source data

We next tested whether PRR-dependent recognition of wild-type R179 or the *dssAB* mutant, which is impaired in interference with PTI-associated root responses, limits bacterial proliferation on roots in mono-associations. Neither the *fls2*, *efr* or *sobir1* single mutant nor the *efr* *fls2* *sobir1* triple mutant showed differential root and shoot colonization by wild-type R179 or the *dssAB* mutant (Fig. 5d and Extended Data Fig. 10a). Moreover, simultaneous application of synthetic flg22 or elf18 peptides with bacteria did not restrict wild-type R179 or *dssAB* mutant growth on wild-type *Arabidopsis* roots (Fig. 5e). Together, this shows that in mono-associations MAMP treatment and PRR-dependent commensal perception do not effectively limit wild-type R179 or *dssAB* mutant root load. To investigate whether the results obtained with R179 have broader relevance within Xanthomonadales, we determined the root load on *Arabidopsis pWER*::*FLS2* plants in mono-associations with 19 root microbiota members derived from *A. thaliana* or *L. japonicus* and two *C. reinhardtii* phycosphere members. The root load of individual Xanthomonadales strains varied markedly by almost 1,000-fold (ranging between 10^6^ and 10^9^ CFU per g root tissue; Extended Data Fig. 10b) and was unchanged upon flg22 treatment for most tested strains. We determined for each strain the difference in root length between flg22- and mock-treated plants in relation to the difference in root length between axenic flg22- and mock-treated plants in relation to its root titre in mono-association (suppressiveness score; Extended Data Fig. 10c). This revealed that Xanthomonadales root load does not correlate with the suppressiveness score and that *Arabidopsis*-derived Xanthomonadales do not colonize their cognate host to higher levels compared with Xanthomonadales strains isolated from other photoautotrophic hosts. Thus, in mono-associations, Xanthomonadales root colonization is determined by strain-specific factors that act dominantly over their conserved immunomodulating activity.

## Discussion

We have shown here that the root commensal *Rhodanobacter* R179, belonging to the deepest branch of the Xanthomonadales, suppresses pattern-triggered immune responses in *Arabidopsis* roots and leaves (Fig. 2a–c). Other Xanthomonadales commensals, recovered from diverse soil-dwelling photoautotrophic eukaryotic hosts, alleviated PTI-associated RGI in *A. thaliana* and *L. japonicus* roots. This shows that immunosuppression is ubiquitous in this core order of the plant microbiota. The near absence of Xanthomonadales in free-living ocean and lake prokaryotic communities, as well as in metazoan guts, supports the notion that the primary reservoirs for this entire order of Gammaproteobacteria are soil and soil-dwelling photoautotrophic hosts^50,51,62,65,84,85^. Hence, maintaining immunomodulatory activities is associated with the prevalence of Xanthomonadales in their primary habitat and community competitiveness in the plant microbiota. The latter conclusion is supported by *Rhodanobacter* R179 *dssAB*-dependent immunomodulation, which gives the commensal a competitive advantage in a bacterial community on *Arabidopsis* roots even in the absence of exogenous stimulation with immunogenic elicitors.

Comparative analysis of immunity-associated responses in wild-type Col-0 and *efr* *fls2* *sobir1* triple mutant plants to live and heat-killed *Rhodanobacter* R179 demonstrated that *Arabidopsis* detects the presence of R179 through the perception of at least three MAMPs. This must be reconciled with our finding that prolonged root colonization in mono-association by live R179 but not heat-killed commensal induced only minor root transcriptional reprogramming (Fig. 2g). Our results suggest that immunoevasion by R179 via the secretion of heat-labile extracellular peptidase(s) for the selective elimination of immunogenic peptides and *dssAB* immunomodulation leads to R179 camouflage. This could partly account for subtle host transcriptional changes induced by R179 colonization despite presenting multiple MAMPs.

The capacity to eliminate or modify immunogenic peptides is unlikely to be R179-specific. Phytopathogenic *Pseudomonas* *syringae* DC3000 releases AprA protease via the type I secretion system to deplete flg22 for full virulence on *Arabidopsis* leaves^71,86^. In addition, Actinobacteria root commensals deplete flg22 via an unknown mechanism^44^. We have shown here that the immunomodulatory root commensal *D. japonica* MF79 eliminates multiple immunogenic peptides (Fig. 4b), similar to R179. This is in line with a recently identified immunosuppressive subtilase A (IssA) in MF79 that cleaves flg22 and AtPep1 (ref. ^87^). This suggests that clearance of immunogenic peptides is conserved in at least two Xanthomonadales genera. We found that peptide clearance is retained in an MF79 T2SS mutant, which is impaired in the suppression of flg22-induced *CYP71A12* defence marker gene expression in roots^43^. This implies that MAMP-eliminating peptidase(s) can be exported by a T2SS-independent mechanism and that *D. japonica* MF79 deploys at least two immunomodulatory mechanisms.

The proposed elimination of flg22 by secreted peptidase(s) for R179 camouflage is counterintuitive as the R179 genome lacks *fliC*, whose product contains the flg22 epitope. Functional diversification of the flg22 epitope has been shown in commensals of the *Arabidopsis* root microbiota, with most peptide variants evading recognition by FLS2 or acting as antagonists of immunogenic flg22^*Pao*^ (refs. ^46,47^). Thus, clearance of immunogenic flg22 epitopes by R179-secreted peptidase(s) does not directly benefit R179 but in principle impacts resident microbiota members expressing *fliC*. This could explain why R179 acts dominantly over the five-member NS3 SynCom containing at least one flg22-presenting *Pseudomonas* commensal to reduce root transcriptomic responses^46^. Hence, R179-secreted peptidase(s) function in hiding the producer and other microbiota members from host recognition. R179-mediated immunosuppression through combined elimination of the DAMP AtPep1 and MAMPs is also in accordance with a recently proposed model for PTI in roots, in which the coincidence detection of cell damage and microbial non-self patterns controls local immune outputs^79^.

To our knowledge, two immunomodulatory mechanisms operating in individual plant commensals, *D. japonica* MF79 and *Rhodanobacter* R179, have not been reported before. MAMP/DAMP elimination by extracellular bacterial peptidase(s) is probably an immunoevasive mechanism, whereas *dssAB*-dependent secretion could be part of an immunoevasive or immunosuppressive strategy. Given that we identified *dssA* and *dssB* in a genetic screen as bacterial determinants for the suppression of PTI-associated RGI in the absence of other commensals and found elevated competitiveness of R179 in SynComs in the root compartment, these bacterial genes probably contribute to host immunosuppression rather than acting directly on other community members. Orthologues of *dssA* and *dssB* are largely conserved in Xanthomonadales (Extended Data Fig. 5d) and may contribute to their success in colonizing plant hosts as a core lineage of the microbiota^51^. The wild-type-like colonization of the R179 *dssAB* mutant on roots and the presence of *dssA* and *dssB* orthologues in the genome of *Rhodanobacter* S772, which fails to colonize roots, argues against a further alternative explanation that these genes act solely as root competence factors. The conservation of *dssA* and *dssB* across the entire order of Xanthomonadales contrasts with the presence of the T3SS, which is restricted to the genus *Xanthomonas* within the Xanthomonadales^58,88^. The T3SS of phytopathogenic *Xanthomonas* species is essential for virulence and was potentially acquired in three independent ancestral acquisition events, followed by multiple sequential losses in commensals^88,89^. As all known T3SS-containing phytopathogenic Xanthomonadales belong to the genus *Xanthomonas*, it is perhaps no coincidence that the opportunistic pathogens L131 and L148 also belong to *Xanthomonas*, despite the absence of the T3SS in these two strains^40,58,59^. L131 also suppressed PTI-associated RGI (Extended Data Fig. 1d,e). Together, these results indicate that T3SS-independent but *dssAB*-dependent immunosuppression is an ancient trait whose evolution preceded the emergence of T3SS-associated pathogen specialization and that is maintained throughout the evolutionary history of Xanthomonadales.

TonB-dependent and ABC transporters frequently function in concert for substrate translocation across the outer and inner membranes of Gram-negative bacteria^90^. TonB-dependent transporters, which are overrepresented in pathogenic *Xanthomonas* genomes, are induced in the genus *Sphingomonas* within the leaf microbiota and are involved in the import of various substrates, including plant-derived carbohydrates, vitamins, and iron and nickel chelates^9,91–93^. Furthermore, TonB-dependent transporters were reported to function in the export of a protease^94^. Future work is needed to identify the dssB substrate(s) involved in the immunomodulatory activity and test the conservation of substrate specificity for dssB orthologues in Xanthomonadales.

The application of immunogenic peptides to *Arabidopsis* or the use of the *efr* *fls2* *sobir1* triple mutant, which barely perceives R179, does not restrict or enhance root colonization by wild-type R179 or the *dssAB* mutant (Fig. 5d,e). The root load of 17 Xanthomonadales commensals in mono-associations with *Arabidopsis* varied 1,000-fold up to 10^9^ CFU g^−1^, a bacterial titre that is comparable to that of phytopathogens, and was not limited by flg22 treatment. By contrast, the proliferation of endophytic *P. syringae* DC3000 pathogen in *Arabidopsis* leaves is restricted by flg22 co-treatment despite T3SS-mediated immunosuppressive activities and AprA protease-mediated flg22 degradation^71,95,96^. The unaltered commensal colonization by Xanthomonadales upon MAMP treatment observed here might result from the plant developmental stage at inoculation—that is, starting from germination—and epiphytic root colonization. In addition, tissue-specific PTI responses might contribute to the apparent uncoupling of Xanthomonadales root load from PTI-mediated bacterial growth restriction demonstrated for pathogenic bacteria.

We have shown that in mono-associations, root colonization by Xanthomonadales commensals is determined by strain-specific factors that act dominantly over their conserved immunomodulating activity. As heterotrophic bacterial proliferation on plants is primarily driven by plant-derived structural and non-structural carbohydrates^8,9^, the strain-specific root load of Xanthomonadales commensals in these mono-associations might reflect diversified host resource use. However, immunomodulation by R179 through *dssAB* was found to exert a dominant influence and elevate R179 root load when the commensal co-colonized with distantly related microbiota members. In this community context, *Rhodanobacter* R179 can be expected to experience enhanced metabolic constraints for root colonization compared with a mono-association. It is conceivable that conserved immunomodulation by Xanthomonadales may function in parallel with or facilitate the diversification of substrate preferences for host resources, thereby contributing to the co-existence of multiple Xanthomonadales strains in the plant microbiota.

## Methods

### Plant cultivation conditions

*A. thaliana* Col-0, *rbohD*^34^, *fls2* SAIL_691-C4 (ref. ^95^), *efr-1* SALK_044334 (ref. ^97^) and *sobir1-12* SALK_050715 (ref. ^98^) and *L. japonicus* Gifu B-129 seeds were obtained from the institute stocks. *A. thaliana pWER*::*FLS2–GFP*^56^ and *pEFR*::NLS–3*mVenus^79^ were provided by N. Geldner, and the *efr-1* *fls2* *sobir1-12* mutant, provided by L. P. Maier and G. Felix (University Tübingen, Germany), was generated by crossing the *efr-1* *fls2* mutant^99^ with the *sobir1-12* mutant. The newly generated mutant line will be made available by the authors on request. The *Arabidopsis efr* *fls2* *rlp1* mutant was present at the University Tübingen, Germany^81^. Plants were cultivated on vertical agar plates under short-day conditions (10 h light, 21 °C; 14 h darkness, 19 °C) with 65% relative air humidity and a light intensity of 120 mE m^−2^ s^−1^. For ethylene measurements, 7-week-old plants grown in potting soil under short-day conditions (8 h light, 24 °C; 16 h darkness, 22 °C) with 40–60% relative air humidity were used.

### Bacterial cultivation conditions

A list of all the bacterial strains used can be found in Supplementary Table 1. Bacteria were obtained from the *At*-SPHERE^5^, *Lj*-SPHERE^55^ and *Cr*-SPHERE^50^ culture collections. MF92 and MF178 were retrieved from Levy et al.^57^. *Xanthomonas campestris* pv. *vesicatoria* 85-10, *Xanthomonas campestris* pv. *campestris* 8004 and *Xanthomonas hortorum* pv. *pelargonii* were provided by U. Bonas (Martin-Luther-University, Halle-Wittenberg, Germany)^100^, L. Noel (lipme Toulouse, France)^101^ and K. Richter (Julius Kühn Institute, Quedlinburg, Germany), respectively. *Xanthomonas arboricola* pv. *pruni* and *Xanthomonas campestris pv. raphani* were present at the University Tübingen (Germany). Bacteria were cultivated in 0.5× TSB (15 g l^−1^ tryptic soy broth, Sigma Aldrich; supplemented, if necessary, with 10 g l^−1^ Bacto Agar, Duchefa Biochemie) at 25 °C with shaking at 180 rpm for liquid cultures only. *E. coli* DH5α λ-pir and pRK600 were cultivated in LB medium. For ethylene measurements, agar-grown *Rhodanobacter* R179 was resuspended (OD_600_ ≈ 1), plated on NYG medium (0.5% (w/v) peptone, 0.3% (w/v) yeast extract, 2% (w/v) glycerol, 1% (w/v) agar) and incubated at 28 °C for 2 days.

### Agar-based cultivation of axenic plants and co-cultivation with bacteria, bacterial supernatants and elicitors

The inoculation of seeds with bacteria was performed as previously described^73^. In brief, *A. thaliana* seeds were surface-sterilized using two 5-min incubations with 70% ethanol and 15 s of incubation in 96% ethanol followed by five to seven washes in H_2_O. Sterilized *A. thaliana* seeds were stratified for 2 days at 4 °C. The surface of *L. japonicus* seeds was abraded using sandpaper, followed by incubation for 20 min in 2% (v/v) bleach; the seeds were then washed five times with H_2_O. Sterilized *L. japonicus* seeds were placed on H_2_O-wetted Whatman paper in square petri dishes and germinated for 4 days under short-day conditions. Saturated overnight bacterial cultures were harvested by centrifugation (11,600 *g*) for 2 min and washed twice in 10 mM MgSO_4_. Bacteria were inoculated into 0.5× Murashige and Skoog (MS) medium (2.22 g l^−1^ MS basal salts medium, Sigma; for testing in-frame mutants, MS medium mod. Mikro-Komplex Phygenera (MD0830); 0.1 g l^−1^ MES, BioChemica; pH 5.7; 10 g l^−1^ Bacto Agar, Duchefa Biochemie) prior to medium solidification at a final concentration of OD_600_ = 0.0005 (per strain for SynComs). The suppression of flg22-induced RGI by wild-type R179 and the mutants varied depending on the agar source and 0.5× MS medium used^102^. We recommend the calibration of plant phenotypes on a given batch of agar and MS medium combination with at least four replicate plates per condition.

Elicitors were also supplemented into the medium prior to solidification at a final concentration of 1 µM. The flg22 (QRLSTGSRINSAKDDAAGLQIA) and AtPep1 (ATKVKAKQRGKEKVSSGRPGQHN) peptides were synthesized by EZbiolab. elf18^R179^ (Ac-AKGKFERTKPHVNVGTIG) and elf18 (Ac-SKEKFERTKPHVNVGTIG) were synthesized by GenScript and EZbiolab, respectively. For inoculation with the bacterial supernatant, bacteria were cultivated overnight in XVM2 minimal medium (20 mM NaCl, 10 mM (NH_4_)_2_SO_4_, 5 mM MgSO_4_, 1 mM CaCl_2_, 0.16 mM KH_2_PO_4_, 0.32 mM K_2_HPO_4_, 0.01 mM FeSO_4_, 10 mM fructose, 10 mM sucrose, 0.03% Casamino acids, pH 6.7)^76^ to a final concentration of OD_600_ = 0.4. Bacteria were pelleted by centrifugation (11,600 *g*, 2 min), and the supernatant was sterile-filtered using filter units with MF-Millipore membrane (0.22 µm; Millex). The supernatant was incubated in boiling water for 30 min for heat treatment. Then, 25 ml of 1× MS with 20 g l^−1^ Bacto Agar was mixed with 25 ml of supernatant prior to medium solidification and poured into square petri dishes. Sterilized seeds were placed on solidified agar in square petri dishes and vertically incubated for 14 days.

### Quantification of bacterial abundance via live colony counts

Roots of 14-day-old seedlings were briefly washed in 10 mM MgSO4, dried with autoclaved Whatman paper and stored in weighed, sterilized screw-cap tubes containing metal beads. The tubes were weighed again to calculate root fresh weights prior to sample homogenization using a tissue homogenizer (Bertin Precellys; 6,200 rpm, 2 × 30 s, 15 s pause in between). Then, 500 µl of 10 mM MgSO_4_ was added, and the samples were homogenized again (same conditions). Dilution series were prepared in 96-well plates, and 10 µl portions were dropped onto 0.5× TSB in square petri dishes and spread across half of the plate by tilting the plate. Bacteria were incubated at 25 °C until colonies could be counted^73^.

### Acute treatment with bacteria or elicitors

Axenic 14-day-old seedlings (see ‘Agar-based cultivation of axenic plants and co-cultivation with bacteria, bacterial supernatants and elicitors’) were submerged in 12 ml of 10 mM MgSO_4_ supplemented with 1 µM elicitor or bacteria, which were directly harvested from 0.5× TSB plates and resuspended in 10 mM MgSO_4_. Bacterial concentration was adjusted to OD_600_ = 1.5, if not indicated differently. For a heat-killed bacterial inoculum, resuspended bacteria were incubated in boiling water for 30 min. After incubation for 5 h on a shaker, roots were directly harvested into screw-cap tubes containing metal beads, frozen in liquid nitrogen and stored at −80 °C until further processing. For co-exposure to flg22 and AtPep1, seedlings were submerged as described for 1 h, residual liquid was removed and the plants were incubated in a cultivation chamber for 4 h prior to harvesting.

### Quantification of defence marker gene expression by RT–qPCR

For reverse transcription quantitative PCR (RT–qPCR), roots of eight to ten plants grown on one square plate were pooled for one sample. Frozen roots were homogenized using a TissueLyser II (QIAGEN, 2 × 29 pps for 40 s) with pre-cooled adaptors. RNA was extracted using the Plant RNA Kit (my-Budget) according to the user manual and eluted in 35 µl of elution buffer. RNA concentrations were measured using a spectrometer (Nanodrop OneC Microvolume UV-Vis Spectrophotometer). Complementary DNA was synthesized from 300–500 ng of RNA using the RevertAid H Minus First Strand cDNA Synthesis Kit (ThermoScientific) in a 20-µl reaction according to the user manual. In brief, 1 µl of oligo(dT)_18_ primer was mixed with RNA in a final volume of 12 µl and incubated at 65 °C for 5 min. Afterwards, 4 µl of 5× Reaction buffer, 2 µl of 10 mM dNTPs, 1 µl of RiboLock RNase Inhibitor (20 U µl^−1^) and 1 µl of RevertAid H Minus M-MuLV Reverse Transcriptase (200 U µl^−1^) were added, and the reaction was incubated at 42 °C for 60 min followed by heat inactivation at 70 °C for 5 min. cDNA was diluted 1:10 prior to qPCR using the iQ SYBR Green Supermix (Bio-Rad). Reactions (20 µl) were prepared with 5 μl of cDNA, 10 μl of SYBR Green Supermix and 0.4 μl of each primer (10 µM; Supplementary Table 6). qPCR was performed using the CFX ConnectTM Real-Time System (Bio-Rad) with the following conditions: 95 °C for 3 min; 95 °C for 15 s, 65 °C for 15 s and 72 °C for 15 s for four cycles; and 95 °C for 15 s, 57 °C for 15 s and 72 °C for 15 s for 39 cycles, followed by a melting curve.

### Confocal microscopy

Six-day-old axenic seedlings cultivated on 0.5× MS agar plates were flush-inoculated with live R179 (OD_600_ = 1.5) and incubated for 1 h with gentle shaking, followed by removal of extensive liquid. The plates were incubated overnight (see ‘Plant cultivation conditions’), and the roots were visualized using confocal microscopy. Confocal laser scanning microscopy was performed on a Zeiss LSM880 inverted confocal scanning microscope. Pictures were taken with an LD C-Apochromat ×40/1.1 water immersion objective. To image root colonization, *Z*-stacks were generated, and maximum-intensity projections were compiled. The following excitation and detection window was used: GFP 488 nm, 493–598 nm.

### 16S rRNA amplicon sequencing and community profiling

After 14 days of co-cultivation of bacteria with *A. thaliana* plants, roots from approximately 20 seedlings grown on three different square plates inoculated with the same bacterial inoculum were pooled, briefly washed in 10 mM MgSO_4_, dried using Whatman paper, transferred into Lysing Matrix E tubes (MP Biomedicals) and frozen in liquid nitrogen. For community profiling upon cultivation in liquid cultures, the indicated SynComs were inoculated in parallel in XVM2 medium (see ‘Bacterial cultivation conditions’) supplemented with 10% TSB at an OD_600_ of 0.05. The next day, bacteria were pelleted by centrifugation (11,600 *g*, 2 min) and frozen in liquid nitrogen. Sample preparation and 16S rRNA community profiling were performed according to Bulgarelli et al.^4^. In brief, roots were homogenized as before (see ‘Quantification of bacterial abundance via live colony counts’), and total DNA was extracted using the FastDNA SPIN Kit for Soil (MP Biomedicals) according to the user manual. DNA was eluted in 80 µl of H_2_O, and concentrations were measured using the Quant-iT PicoGreen dsDNA Assay (Thermo Fisher Scientific) and adjusted to a concentration of 3.5 ng µl^−1^.

PCR I and II were both performed in 25-µl reaction volumes containing 10.5 ng of sample DNA, 2 U of DFS-Taq DNA polymerase (Bioron), 1× incomplete buffer, 2 mM MgCl_2_, 0.3% bovine serum albumin, 0.2 mM dNTPs (Life Technologies) and 0.3 μM forward and reverse primers. PCR I was performed in triplicates with the primers 799F and 1192R and an additional 0.004 ng of pBCC23 plasmid DNA per reaction (Supplementary Table 6) using the following conditions: 94 °C for 2 min; 94 °C for 30 s, 55 °C for 30 s and 72 °C for 30 s for 25 cycles; and a final extension at 72 °C for 10 min. Afterwards, 20 µl of the pooled triplicates of PCR I were purified by primer and protein digestion with 5 U of Antarctic phosphatase, 20 U of exonuclease I and 1× Antarctic phosphatase buffer (New England Biolabs) at 37 °C for 30 min, followed by enzyme deactivation at 85 °C for 15 min. The reactions were centrifuged for 15 min (4,200 *g*), and 3 μl of supernatant was used for PCR II. In PCR II, the samples were double-indexed with one indexed forward primer per full factorial replicate and an indexed reverse primer for each sample per full factorial replicate. PCR reactions and conditions were similar to those for PCR I, but the cycles were reduced to ten. Afterwards, the PCR quality and quantity were checked on a 1.5% agarose gel, indexed amplicons of biological replicates were pooled and separated from plant 16S rRNA amplicons on a 1.5% agarose gel and bacterial 16S rRNA was cut out together with the pBCC23 amplicon and purified using the QIAquick Gel Extraction Kit (QIAGEN) according to the user manual. DNA concentrations were again measured using the Quant-iT PicoGreen dsDNA Assay (Thermo Fisher Scientific), and equal amounts of DNA were pooled. The library was then again purified and concentrated using Agencourt AMPure XP beads, and the DNA concentration was determined using the Quantus Fluorometer (Promega). Finally, 17.5 ng µl^−1^ of the final library were subjected to paired-end Illumina sequencing in-house, using the MiSeq sequencer and custom sequencing primers.

Sequencing reads were demultiplexed according to the respective index sequence and quality-filtered using the QIIME pipeline^103^. Then, forward and reverse sequencing reads were merged using flash2 software^104^ and aligned to reference *V5V7* 16S rRNA sequences of all SynCom members and the pBCC23-specific amplicon sequence using Rbec^105^. For experiments with S2 and NS4, reads were manually curated for the inoculated strains for each sample. Finally, an amplicon sequence variant table was generated. First, counts were normalized by the pBCC23 spike in counts for each sample. Bray–Curtis dissimilarities were calculated after in silico depletion of R179-specific counts using the vegdist function of the vegan package (v2.5.7)^106^, and the constrained principal component analysis was performed using the vegan capscale function constraining by the interaction between the R179 derivative (wt/*dssAB*) and SynCom (suppressive/non-suppressive) and conditioning by technical and biological replicates. Finally, all amplicon data were visualized using ggplot2 (ref. ^107^) implemented in the tidyverse package (v.1.3.1)^108^.

### Quantification of peptides by mass spectrometry

Bacterial supernatants were prepared as described in ‘Agar-based cultivation of axenic plants and co-cultivation with bacteria, bacterial supernatants and elicitors’. Here, additionally, the peptides dRGF1 (DYSNPGHHP-Hyp-RHN) and PSY1 (D-Y(SO_3_H)-GDPSANPKHDPGV-Hyp-Hyp-S) synthesized by Scilight Peptide were incubated for 1 h with 1 µM of peptides in a plant cultivation light chamber (day-light condition; see ‘Plant cultivation conditions’), and the samples were frozen at −20 °C until further processing. For the investigation of AtPep1 degradation, the AtPep1 peptide was first modified by acetylation (six to seven times). To this end, 100-µl aliquots of peptides were treated with 10 µl of NHS acetate solution (1 M NHS acetate in 50% ACN/PBS) and incubated for 30 min with shaking. The reaction was quenched by the addition of 100 µl of 100 mM Tris-HCl (pH 8.5) and incubated for 10 min. For comparison of the non-acetylated peptides with the above-described modified AtPep1 peptides, aliquots of the non-acetylated peptides were processed as described above, but without the modifying NHS reaction. To this end, 100-µl aliquots of the sample were mixed with 100 µl of PBS buffer (137 mM NaCl, 2.7 mM KCl, 10 mM Na_2_HPO_4_, 1.8 mM KH_2_PO_4_; pH 7.4). The samples were then further diluted with 100 µl of 100 mM Tris-HCl (pH 8.5) and incubated for 10 min. All samples were acidified with 5 µl of TFA and desalted using StageTips with C18 Empore disk membranes (3M)^109^. The samples were dried in a vacuum evaporator and dissolved in 10 µl of 2% ACN (0.1% TFA for analysis).

The samples were analysed using an EASY-nLC 1200 (Thermo Fisher) coupled to a QExactive Plus mass spectrometer (Thermo Fisher). Peptides were separated on 16-cm frit-less silica emitters (New Objective, 75 µm inner diameter), packed in-house with reversed-phase ReproSil-Pur C18 AQ 1.9 µm resin (Dr. Maisch). Peptides were loaded on the column and eluted for 50 min using a segmented linear gradient of 5% to 95% solvent B (0 min, 5% B; 0–5 min, 15% B; 5–35 min, 50% B; 35–45 min, 95% B; 45–50 min, 95% B) (solvent A: 0% ACN, 0.1% FA; solvent B: 80% ACN, 0.1% FA) at a flow rate of 300 nl min^−1^. Mass spectra were acquired in data-dependent acquisition mode with a TOP10 method. Mass spectrometry spectra were acquired in the Orbitrap analyser with a mass range of 300–1,500 *m*/*z* at a resolution of 70,000 full width at half maximum (FWHM) and a target value of 3 × 10^6^ ions. Precursors were selected with an isolation window of 1.3 *m*/*z*. Higher-energy collision dissociation (HCD) fragmentation was performed at a normalized collision energy of 25. Tandem mass spectrometry (MS/MS) spectra were acquired with a target value of 5 × 10^5^ ions at a resolution of 17,500 FWHM, a maximum injection time of 85 ms and a fixed first mass of 100 *m*/*z*. Peptides with a charge of 1 or greater than 6 or with an unassigned charge state were excluded from fragmentation for MS/MS; dynamic exclusion for 20 s prevented repeated selection of precursors. The raw data were analysed on the MS1 level using Skyline (https://skyline.ms)^110^, and the results were filtered for the respective intact peptides, or the acetylated version in the case of AtPep1. Peaks were checked and, if needed, integrated manually. Finally, peak areas were exported for further processing.

### Root proteomics by mass spectrometry

#### Sample preparation

Approximately 20 mg of root tissue from 14-day-old seedlings co-cultivated with R179 on three square plates was harvested into Lysing Matrix E tubes (MP Biomedicals) and immediately frozen in liquid nitrogen. Frozen root samples were disrupted using a Retsch mill at 50 Hz for 5 min. Then, 350 µl of hot SDT extraction buffer (4% SDS in 100 mM Tris-HCl, pH 8.5, 0.1 M DDT) was added, and the samples were incubated at 95 °C for 10 min. Next, the samples were centrifuged for 5 min at 5,000 *g*, and the supernatants were again centrifuged at full speed for 10 min. Protein concentrations were determined using a Pierce 660 mn protein assay, and aliquots corresponding to 50 µg of total protein were digested using a filter-aided sample preparation method^111^. Briefly, 450-µl extracts were loaded onto a Vivacon 500 (Sartorius Stedim Biotech), centrifuged at 14,000 *g* and washed once with 450 µl of UA (8 M in 100 mM Tris-HCl, pH 8.5). Next, the samples were alkylated with CAA (100 µl, 55 mM CAA) for 20 min at room temperature in the dark. After centrifugation at 14,000 *g* for 10 min, the samples were washed three times with UA (450 µl), and filters were exchanged onto fresh tubes to collect flow-through for the following steps. Next, 50 µl of LysC solution (1:100 enzyme/protein in 100 mM Tris-HCl, pH 8.5) was added, and the samples were incubated for 3 h at room temperature. Then, 250 µl of trypsin solution (1:100 enzyme/protein in 100 mM Tris-HCl, pH 8.5) was added onto the filter, the filter was filled with 100 mM Tris-HCl pH 8.5 to the mark, the solutions were mixed well and the samples were incubated overnight at 37 °C. After digestion, the filters were centrifuged to collect the flow-through and were washed once with 50 µl of 100 mM Tris-HCl, pH 8.5. The flow-throughs were combined and acidified/desalted as described in ‘Quantification of peptides by mass spectrometry’. Peptide concentrations were adjusted to 0.2 µg µl^−1^.

#### Data acquisition

The samples were analysed using an Ultimate 3000 RSLC nano (Thermo Fisher) coupled to an Orbitrap Exploris 480 mass spectrometer equipped with an FAIMS Pro interface for field asymmetric ion mobility separation (Thermo Fisher). Peptides were pre-concentrated on an Acclaim PepMap 100 pre-column (75 µm × 2 cm, C18, 3 µM, 100 Å, Thermo Fisher) using the loading pump and buffer A** (water, 0.1% TFA) with a flow of 7 µl min^−1^ for 5 min. Peptides were separated as described in ‘Quantification of peptides by mass spectrometry’. Peptides were loaded on the column and eluted for 130 min using a segmented linear gradient of 5% to 95% solvent B (0–5 min, 5% B; 5–65 min, 20% B; 65–90 min, 35% B; 90–100 min, 55% B; 100–105 min, 95% B; 105–115 min, 95% B; 115–115.1 min, 5% B; 115.1–130 min, 5% B) (solvent A: 0% ACN, 0.1% FA; solvent B: 80% ACN, 0.1% FA) at a flow rate of 300 nl min^−1^. Mass spectra were acquired in data-dependent acquisition mode with a TOP_S method using a cycle time of 2 s. For field asymmetric ion mobility separation, two compensation voltages (−45 and −60) were applied with a cycle time of 1 s for each experiment. Mass spectrometry spectra were acquired in the Orbitrap analyser with a mass range of 320–1,200 *m*/*z* at a resolution of 60,000 FWHM and a normalized AGC target of 300%. Precursors were filtered using the MIPS option (MIPS mode = peptide), the intensity threshold was set to 5,000 and precursors were selected with an isolation window of 1.6 *m*/*z*. Higher-energy collision dissociation fragmentation was performed at a normalized collision energy of 30%. MS/MS spectra were acquired with a target value of 75% ions at a resolution of 15,000 FWHM, inject time set to auto and a fixed first mass of 120 *m*/*z*. Peptides with a charge of +1 or greater than 6 or with an unassigned charge state were excluded from fragmentation for MS^2^.

#### Data analysis

The raw data were processed using MaxQuant software (v.1.6.3.4, http://www.maxquant.org/)^112^ with label-free quantification (LFQ) and iBAQ enabled^113^. MS/MS spectra were searched using the Andromeda search engine against an *A. thaliana* database (TAIR10_pep_20101214; ftp://ftp.arabidopsis.org/home/tair/Proteins/TAIR10_protein_lists/) and sequences of 248 common contaminant proteins and decoy sequences. Trypsin specificity was required, and a maximum of two missed cleavages were allowed. Minimal peptide length was set to seven amino acids. Carbamidomethylation of cysteine residues was set as fixed, and oxidation of methionine and protein amino-terminal acetylation were set as variable modifications. The match-between-runs option was enabled. Peptide-spectrum-matches and proteins were retained if they were below a false discovery rate of 1% in both cases. Statistical analysis of the MaxLFQ values was carried out using Perseus (v.1.5.8.5, http://www.maxquant.org/). Quantified proteins were filtered for reverse hits, and hits ‘identified by site’ and MaxLFQ values were log_2_-transformed. Quantified proteins were grouped by condition, and only those hits were retained that had three valid values in one of the conditions. Missing values were imputed from a normal distribution (1.8 downshift, separately for each column).

### PacBio genome sequencing, assembly and analysis

Genomic DNA was isolated using the Macherey-Nagel Bacteria DNA Kit, and the quality of the extracted DNA was assessed with FEMTOpulse and Agilent technologies. A long-insert *Tn5* library was then prepared following the LongPlex Long Fragment Multiplexing Kit (seqWell) and pooled before sequencing on the PacBio Revio platform at the Max Planck Genome Centre Cologne. PacBio reads were filtered and subjected to quality control using Filtlong v.0.2.1 (available at https://github.com/rrwick/Filtlong), applying the following parameters: min_length, 1,000; keep_percent, 90; target_bases, 500,000,000. The filtered reads were subsequently assembled with Flye v.2.9.5-b1801 (ref. ^114^) using parameters that included the following: genome-size, 4 m; asm-coverage, 70; iterations, 4. To ensure accurate start positions for the assembled genomes, the assembly was anchored at the dnaA gene with dnaapler v.0.8.1 (ref. ^115^). Finally, annotations for the assembled genomes were generated using Bakta v.1.9.4 (ref. ^116^).

In-frame deletions and *mini-Tn5* insertions were confirmed through BLASTP v.2.16.0 (ref. ^117^) using the default parameters, comparing the wild-type and mutant R179 assembled genomes against the amino acid sequences of dssA and dssB. Further verification of the mutations was conducted via multiple genome alignment using the progressiveMauve algorithm, implemented in Mauve v.2015-02-25 (ref. ^118^), also with the default parameters. The results from Mauve were manually inspected to exclude any unintended structural rearrangements within the chromosome.

### Phylogenetic trees

We compiled a set of 1,765 genomes including all genomes classified as Xanthomonadales found in the NCBI database^119^ as of October 2019, as well as the available genomes from isolated *Arabidopsis* strains from the United States and the *Cr*-, *At*- and *Lj*-SPHERE culture collections^5,50,55,57^. We also selected 20 *P. syringae* genomes from the NCBI database as an outgroup. We searched within the final set of 1,785 genome assemblies for the presence of 31 conserved, single-copy marker genes, known as AMPHORA genes^120^, using HMMER (v.3.3.1)^121^. Next, the sequences of each of the 31 genes were aligned separately using Clustal Omega (v.1.2.0)^122^. The alignments for each AMPHORA gene were concatenated into continuous alignments, and missing sequences were filled with alignment gaps. On the basis of this concatenated alignment, a maximum likelihood phylogeny was calculated using FastTree (v.2.1.3)^123^ and sequentially rooted with the *Pseudomonas* outgroup. The rooted tree was visualized using the Interactive Tree of Life web tool^124^. We predicted orthologous groups of R179 *dssA* and *dssB* from protein-coding regions of genomes from the *At*-R-SPHERE and a selection of Xanthomonadales strains (Supplementary Table 7) using Orthofinder (v.2.3.7, default parameters)^125^. The presence of orthologues of *dssA* and *dssB* in all publicly available Xanthomonadales genomes was investigated by querying the coding sequences of *dssA* and *dssB* against the coding sequences of 1,772 Xanthomonadales genomes using BLASTX v2.12.0 (ref. ^117^). Percent identity value > 30 and *e* < 1 × 10^−10^ were used as thresholds for true orthologue prediction. The phylogenetic tree was generated as before using AMPHORA marker genes and visualized using the ggtree package (v3.1.4) in R (v4.0.5)^126^. Species, which were members of the orthogroups, were marked on the phylogenetic tree. All software used was executed with the default parameters.

### Distribution of reference Xanthomonadales in the MAP

We queried the MAP^127^ for Operational Taxonomic Units (OTUs) clustered at 99% identity (based on the full-length 16S rRNA gene) that belonged to the order Xanthomonadales. We then selected reference OTUs, which we defined as containing a reference genome and having a defined species as well as a type strain. This led to a set of 57 OTUs (40 belonging to the Xanthomonadaceae family and 17 to the Rhodanobacteraceae family). For each of these OTUs, we downloaded the associated abundance table from the MAP website (https://microbeatlas.org/). We grouped the samples by environment type (animal, aquatic, plant, soil and unknown) and, for each OTU, normalized the number of samples from each environment type by the total number of samples from that environment included in the MAP. We then computed the normalized proportion of samples belonging to soil and plant-associated environments for each OTU (Supplementary Table 8).

### Distribution of Xanthomonadales MAGs in aquatic, terrestrial and host-associated environments

To estimate the ecological distribution of potentially uncultivated Xanthomonadales lineages, we searched large-scale metagenomic datasets for MAGs belonging to that order, including soil (40,039 MAGs spanning 21,076 species from 3,304 globally distributed soil metagenomes)^60^, leafy greens (910 MAGs from 107 metagenomes across three host species)^61^, lake (1,184 MAGs spanning 1,008 species from 308 Canadian lakes)^62^, marine (26,309 MAGs spanning 5,098 species from 1,028 globally distributed, mostly free-living, ocean microbial communities)^65^, rumen (4,941 MAGs across 283 cattle)^63^ and human (287,002 MAGs spanning 4,645 species from over 10,000 metagenomes)^64^ datasets. For each of these datasets, we selected MAGs belonging to the Xanthomonadales order on the basis of the Genome Taxonomy Database^128^ annotations provided with the release of the dataset. Whenever possible, the number of Xanthomonadales MAGs was also translated into species-level units as defined by the original dataset (Supplementary Table 9).

### Establishment of an R179 *mini-Tn5* mutant library

R179, *E. coli* carrying pUTmTn*5*Km2 (ref. ^72^) and the conjugation-competent helper *E. coli* PRk600 were mixed in a 2:1:1 ratio and co-cultivated overnight on 0.5× TSB plates at 25 °C. The conjugation mix was resuspended in 0.5× TSB, supplemented with 20% glycerol and frozen at −80 °C. Subsequently, aliquots of the conjugation mix were plated on 0.5× TSB plates supplemented with 50 µg ml^−1^ kanamycin and 100 µg ml^−1^ nitrofurantoin and incubated at 25 °C for 48 h. Afterwards, single colonies were inoculated into 100 µl of 0.5× TSB (same antibiotics) in 96-well culture plates, incubated at 25 °C and 180 rpm for 48 h and immediately frozen at −80 °C after adding 20% glycerol.

### High-throughput screening for the loss of flg22-induced RGI

Phytostrip-based high-throughput screening of R179 *mini-Tn5* mutants was performed as previously described^73^. In brief, R179 *mini-Tn5*-transposon mutants were inoculated in 0.5× TSB in 96-deep-well plates and incubated at 25 °C and 180 rpm for 48 h. Bacteria were pelleted by centrifugation (10 min, 4,200 *g*) and washed twice with 10 mM MgSO_4_. After the last centrifugation, bacterial growth was qualitatively documented by scanning 96-deep-well plates. Washed bacteria were then resuspended in 0.5× MS medium (see ‘Agar-based cultivation of axenic plants and co-cultivation with bacteria, bacterial supernatants and elicitors’) to reach a bacterial concentration of approximately OD_600_ = 0.015. Afterwards, sealed phytostrips were filled with 100 µl of resuspended bacterial culture. Then, 0.5× MS (2.22 g l^−1^ MS basal salts, Sigma; 0.1 g l^−1^ MES, BioChemica; pH 5.7; 1.2% (w/v) Phytagel, Sigma-Aldrich) was supplemented with 1 µM flg22 (see ‘Agar-based cultivation of axenic plants and co-cultivation with bacteria, bacterial supernatants and elicitors’), and 230 µl was added to the resuspended bacteria in the phytostrips. Phytostrip-compatible 96-deep-well plates were filled with liquid 0.5× MS medium, and the phytostrips were placed on top after the medium solidified. Finally, five sterilized *A. thaliana pWER*::*FLS2–GFP* seeds were added to each well, and the assembled system was placed into an autoclaved rectangular box and incubated for 2 to 3 weeks (see ‘Plant cultivation conditions’). Root length was documented by scanning and qualitatively scored.

### Identification of *mini-Tn5* insertion sites

Chromosomal *mini-Tn5* transposon integration sites were determined similarly as described before using TAIL PCR^72,129^. The first PCR was conducted using the primers JO4/JO28, 10 ng of gDNA (see ‘Targeted mutagenesis’) and the DFS-Taq DNA polymerase (Bioron) using the following conditions: 94 °C for 2 min; 94 °C for 30 s, 30 °C for 30 s and 72 °C for 1 min for 6 cycles; 94 °C for 30 s, 45 °C for 30 s and 72 °C for 1 min for 30 cycles; and a final extension at 72 °C for 10 min. For the second PCR, a 1:10 dilution of the PCR product was used with the JO1/JO5 primers. The following conditions were used: 94 °C for 2 min; 94 °C for 30 s, 57 °C for 30 s and 72 °C for 1 min for 30 cycles; and a final extension at 72 °C for 10 min. Finally, the PCR products were separated using gel electrophoresis, and the most prominent band was extracted and Sanger-sequenced with the JO1 primer (Eurofins Scientific). The sequencing results were analysed in CLC Main Workbench (QIAGEN). A list of the oligonucleotides used can be found in Supplementary Table 6.

### Targeted mutagenesis

Targeted mutants were generated using the Golden Gate-compatible pOGG2 vector^130^. In brief, R179 DNA was extracted by resuspending a bacterial colony in 25 µl of buffer I (25 mM NaOH, 0.2 mM EDTA, pH 12) followed by incubation at 95 °C for 30 min and the addition of 25 µl of buffer II (40 mM Tris-HCl). Approximately 750-nucleotide sequences flanking *dssA* and *dssB* upstream and downstream were amplified using the primers JO105/JO106 and JO107/JO108 and JO184/JO185 and JO186/JO195, respectively (Phusion Hot Start High-Fidelity DNA polymerase; Thermo Scientific, according to the user manual). PCR products were assembled into pOGG2 by a restriction/ligation reaction with BsaI (Thermo Scientific) and T4 DNA ligase (New England Biolabs) as previously described^131^. Colony PCR-positive plasmids (DFS-Taq DNA polymerase; Bioron, primer JO127/128)) were extracted using a NucleoSpin Plasmid kit (Macherey-Nagel), and the final vectors were validated by Sanger sequencing (Eurofins Scientific). The sequencing results were analysed in CLC Main Workbench (QIAGEN). Vectors were integrated by R179 conjugation with the respective pOGG2 derivate harbouring *E. coli* DH5α λ-pir and the *E. coli* helper PRk600. The conjugation mix was incubated at 25 °C overnight, and transformants/recombinants were identified by sequential selection on 0.5× TSB supplemented with spectinomycin (25 µg ml^−1^), nitrofurantoin (100 µg ml^−1^) and 10% sucrose. The R179 mutants were validated using the primers JO147/148 for the deletion of *dssA* and the primers JO182/JO183 for the deletion of *dssB* followed by Sanger sequencing.

### RNA sequencing for root transcriptomics

#### Sample preparation and sequencing

The roots of approximately 30 seedlings cultivated on three square plates were pooled for one sample, briefly washed in 10 mM MgSO_4_, dried using Whatman paper and immediately frozen in liquid nitrogen in Lysing Matrix E tubes (MP Biomedicals). The roots were homogenized using a TissueLyser II (QIAGEN, 2 × 29 pps for 40 s) with pre-cooled adapters. RNA was extracted using the RNeasy Plant Mini Kit (QIAGEN) according to the user manual with optional on-column DNase digestion, and RNA concentrations were measured using a spectrometer (Nanodrop OneC Microvolume UV-Vis Spectrophotometer). Library preparation and sequencing were conducted at Novogene (Cambridge, UK) using the Illumina HiSeq2500 platform. Approximately 10 million paired-end reads were obtained per sample.

#### Analysis of the raw data

The quality of the raw FASTQ files was assessed using fastQC (http://www.bioinformatics.babraham.ac.uk/projects/fastqc). Quality-controlled files were mapped to the *A. thaliana* reference genome version TAIR10 (ref. ^132^) using STAR (v.2.7.10b)^133^. The aligned read counts were obtained with featureCounts in the R package Rsubread (v.2.12.0)^134^. The R package sva (v.3.46.0)^135^ was used to account for hidden batch effects. Using the function sva, the number of surrogate variables was determined to be 6. Differential analysis was performed using the R package DESeq2 (v.1.38.0)^136^. To reduce the number of false positive DEGs, only the genes that had DESeq2 normalized counts ≥10 in at least three samples were analysed. The thresholds for DEGs were set to |log_2_(fold change)| ≥ 1 and false-discovery-rate-corrected *P* ≤ 0.05. The functional annotations of genes based on Araport11 annotations^137^ were downloaded from the TAIR website (https://www.arabidopsis.org/).

#### Clustering and GO term enrichment analysis

The clustering of DEGs was performed by taking the raw log_2_(fold change) values with hierarchical clustering, using the euclidean distance and ward.D method as parameters^138^. The number of optimal clusters was identified by using the Silhouette score^139^ calculated by the R package NbClust v.3.0.1 (ref. ^140^). The R package ComplexHeatmap (v.2.14.0)^141^ was used for visualization. The GO term enrichment analyses of the gene clusters were performed using the R package topGO (v.2.50.0)^142^ with the enrichment threshold of *P* = 0.01. The significance of the overlaps between clusters from this study and from the previous study^44^ were assessed by a one-sided Fisher’s exact test, with the threshold of false-discovery-rate-adjusted *P* = 0.05.

### Preparation of microbial extracts and ethylene measurement

Bacteria incubated for 2 days were resuspended at a concentration of 10% (w/v) in ice-cold water. Bacterial cells were pelleted by centrifugation at 4,500 *g* and 4 °C for >30 min, washed with ice-cold water and lysed by sonication (1 kJ ml^−1^ over 20 min) on ice. Cell debris was removed by centrifugation at >14,000 *g* and 4 °C for 20 min. The supernatant was used either without treatment or after heat treatment at 95 °C for 30 min. Serial dilutions of native and heat-treated lysates were prepared in 1% (w/v) BSA and 100 mM NaCl. The same solution without lysate was used for mock treatments.

Ethylene biosynthesis was measured as previously described^143^. In brief, *A. thaliana* leaves were cut into square pieces (4 × 4 mm^2^) and floated overnight on water. Three pieces each were then transferred to 6-ml glass tubes containing 500 µl of water. After bacterial lysates (or AtPep1) were added, the reaction tubes were sealed with rubber stoppers and agitated on a shaker at 100 *g*. At 5 h post treatment, 1 ml of head-space volume was sampled and subjected to gas chromatographic analysis (GC 14 A, by Shimadzu). The gas sample was separated on an alumina column (1.2 m × 3 mm, packed with Al_2_O_3_ grains) via isocratic elution at 140 °C and with a constant N_2_ carrier gas stream (250 kPa). Signals were detected with an FID (50 kPa of H_2_, 25 kPa of air). Ethylene peaks were identified according to their retention time, integrated and quantified by comparison to an analytical standard.

### Reporting summary

Further information on research design is available in the Nature Portfolio Reporting Summary linked to this article.

## Supplementary information

Reporting Summary

Supplementary Tables Supplementary Tables 1–11.

## Source data

Source Data Fig. 1 Statistical source data for Fig. 1.

Source Data Fig. 2 Statistical source data for Fig. 2.

Source Data Fig. 3 Statistical source data for Fig. 3.

Source Data Fig. 4 Statistical source data for Fig. 4.

Source Data Fig. 5 Statistical source data for Fig. 5.

Source Data Extended Data Fig. 1 Statistical source data for Extended Data Fig. 1.

Source Data Extended Data Fig. 3 Statistical source data for Extended Data Fig. 3.

Source Data Extended Data Fig. 4 Statistical source data for Extended Data Fig. 4.

Source Data Extended Data Fig. 5 Statistical source data for Extended Data Fig. 5.

Source Data Extended Data Fig. 6 Statistical source data for Extended Data Fig. 6.

Source Data Extended Data Fig. 7 Statistical source data for Extended Data Fig. 7.

Source Data Extended Data Fig. 8 Statistical source data for Extended Data Fig. 8.

Source Data Extended Data Fig. 9 Statistical source data for Extended Data Fig. 9.

Source Data Extended Data Fig. 10 Statistical source data for Extended Data Fig. 10.

## Data Availability

The raw 16S rRNA amplicon reads, RNA sequencing reads and PacBio genome assemblies have been deposited in the European Nucleotide Archive at EMBL-EBI under accession number PRJEB79854. The root proteome data are available at the Proteomics Identification Database at EMBL-EBI under accession number PXD045054. Mass spectrometry data for peptide quantification have been submitted to the Panorama data repository (https://panoramaweb.org/rootmicrobiota.url). Proteomics MS/MS spectra were searched against the *Arabidopsis* database TAIR10_pep_20101214 (ftp://ftp.arabidopsis.org/home/tair/Proteins/TAIR10_protein_lists/). For functional annotations of *Arabidopsis* transcripts, annotations from Araport11 were used (https://www.arabidopsis.org/). Publicly available genomes used for meta-analysis are listed in the supplementary tables. Source data are provided with this paper.

## Code availability

The scripts used to generate individual figure panels are available via GitHub at https://github.com/OrdonJ/Xanthomonadales_immunosuppression_R179.
