# Supplementary material for: Conserved immunomodulation and variation in host association by Xanthomonadales commensals in Arabidopsis root microbiota
Source: Nat Plants. 2025 Feb 19;11(3):612–31. doi: 10.1038/s41477-025-01918-w (PMC11928319; doi:10.1038/s41477-025-01918-w)
Supplement: Supplementary file 1 — Reporting Summary [file 41477_2025_1918_MOESM1_ESM.pdf]

nature portfolio

Corresponding author(s): Paul Schulze-Lefert

Last updated by author(s): Dec 16, 2024

Reporting Summary

Nature Portfolio wishes to improve the reproducibility of the work that we publish. This form provides structure for consistency and transparency in reporting. For further information on Nature Portfolio policies, see our Editorial Policies and the Editorial Policy Checklist.

Statistics

For all statistical analyses, confirm that the following items are present in the figure legend, table legend, main text, or Methods section.

n/a	Confirmed	
☐	☒	The exact sample size (*n*) for each experimental group/condition, given as a discrete number and unit of measurement
☐	☒	A statement on whether measurements were taken from distinct samples or whether the same sample was measured repeatedly
☐	☒	The statistical test(s) used AND whether they are one- or two-sided<br>*Only common tests should be described solely by name; describe more complex techniques in the Methods section.*
☒	☐	A description of all covariates tested
☐	☒	A description of any assumptions or corrections, such as tests of normality and adjustment for multiple comparisons
☐	☒	A full description of the statistical parameters including central tendency (e.g. means) or other basic estimates (e.g. regression coefficient) AND variation (e.g. standard deviation) or associated estimates of uncertainty (e.g. confidence intervals)
☐	☒	For null hypothesis testing, the test statistic (e.g. *F*, *t*, *r*) with confidence intervals, effect sizes, degrees of freedom and *P* value noted<br>*Give P values as exact values whenever suitable.*
☒	☐	For Bayesian analysis, information on the choice of priors and Markov chain Monte Carlo settings
☒	☐	For hierarchical and complex designs, identification of the appropriate level for tests and full reporting of outcomes
☒	☐	Estimates of effect sizes (e.g. Cohen's *d*, Pearson's *r*), indicating how they were calculated

Our web collection on statistics for biologists contains articles on many of the points above.

Software and code

Policy information about availability of computer code

Data collection	16s rRNA libraries were subjected to paired-end Illumina sequencing in-house, using the MiSeq sequencer and custom sequencing primers.<br>Peptides were quantified using an EASY-nLC 1200 (Thermo Fisher) coupled to a QExactive Plus mass spectrometer (Thermo Fisher) as described in the method section.<br>Protein samples were analyzed using an Ultimate 3000 RSLC nano (Thermo Fisher) coupled to an Orbitrap Exploris 480 mass spectrometer equipped with a FAIMS Pro interface for Field asymmetric ion mobility separation (Thermo Fisher).<br>Bacterial genome sequencing was performed using the Pacific Biosciences Revio platform.<br>RNA sequencing was conducted at Novogene using the Illumina HiSeq2500 platform.
Data analysis	16S rRNA sequencing reads were demultiplexed and filtered using the QIIME pipeline (v2 2021.2). Reads were merged using flash2 software (V.2.2.00) and assigned to reference sequences using Rbec (v1.8.0). For further analysis the vegan (v2.5.7) and tidyverse (v1.3.1) packages in R were used. Raw peptide quantities were analyzed on MS1 level using Skyline (https://skyline.ms), results were filtered for the respective intact peptides, or the acetylated version in the case of AtPep1. Raw proteomics data were processed using MaxQuant software (v1.6.3.4, http://www.maxquant.org/) with label-free quantification (LFQ) and iBAQ enabled as further described in the method section. For phylogenetic trees, AMPHORA genes were queried using HMMER (v3.3.1). Sequences were aligned using Clustal Omega (v1.2.0). The maximum likelihood phylogeny was calculated using FastTree (v2.1.3). PacBio reads were filtered and subjected to quality control using Filtlong v0.2.1. The filtered reads were subsequently assembled with Flye (v2.9.5-b1801). The assembly was anchored at the dnaA gene with dnaapler (v0.8.1). Annotations for the assembled genomes were generated using Bakta v1.9.4. RNA seq data were assessed using fastQC (http://www.bioinformatics.babraham.ac.uk/projects/fastqc), quality-controlled using STAR (v2.7.10b) and aligned with featureCounts in R package Rsubread (v2.12.0). Hidden batch effects were accounted for using the R sva (v3.46.0) package and differential analysis was performed by using the R package DESeq2 (v1.38.0). Number of optimal clusters were calculated by the R package NbClust (v3.0.1). R package ComplexHeatmap (v2.14.0) was used for visualization. GO term enrichment analyses of the gene clusters were performed by R package

topGO (v2.50.0). Data analysis was performed using RStudio (v2022.07.0) running R version 4.0.5. Customized code is available at Github (https://github.com/OrdonJ/Xanthomonadales_immunosuppression_R179).

For manuscripts utilizing custom algorithms or software that are central to the research but not yet described in published literature, software must be made available to editors and reviewers. We strongly encourage code deposition in a community repository (e.g. GitHub). See the Nature Portfolio guidelines for submitting code & software for further information.

Data

Policy information about availability of data

All manuscripts must include a data availability statement. This statement should provide the following information, where applicable:

- Accession codes, unique identifiers, or web links for publicly available datasets
- A description of any restrictions on data availability
- For clinical datasets or third party data, please ensure that the statement adheres to our policy

Raw 16S rRNA amplicon reads, RNA sequencing reads and PacBio genome assemblies have been deposited in the European Nucleotide Archive (ENA) at EMBL-EBI under accession number PRJEB79854 (https://www.ebi.ac.uk/ena/browser/view/PRJEB79854). Root proteome data are available at the Proteomics Identification Database (PRIDE) at EMBL-EBI under accession number PXD045054 (http://www.ebi.ac.uk/pride/archive/projects/PXD045054). Mass spectrometry data for peptide quantification have been submitted to the Panorama data repository (https://panoramaweb.org/rootmicrobiota.url). Additional source data are published alongside the manuscript. Publicly available genomes used for meta-analysis are listed in supplementary tables. Proteomics MS/MS spectra were searched against the Arabidopsis database TAIR10_pep_20101214 (ftp://ftp.arabidopsis.org/home/tair/Proteins/ TAIR10_protein_lists/). For functional annotations of Arabidopsis transcripts annotations from Araport11 were used (https://www.arabidopsis.org/).

Research involving human participants, their data, or biological material

Policy information about studies with human participants or human data. See also policy information about sex, gender (identity/presentation), and sexual orientation and race, ethnicity and racism.

Reporting on sex and gender	N/A
Reporting on race, ethnicity, or other socially relevant groupings	N/A
Population characteristics	N/A
Recruitment	N/A
Ethics oversight	N/A

Note that full information on the approval of the study protocol must also be provided in the manuscript.

Field-specific reporting

Please select the one below that is the best fit for your research. If you are not sure, read the appropriate sections before making your selection.

☒ Life sciences ☐ Behavioural & social sciences ☐ Ecological, evolutionary & environmental sciences

For a reference copy of the document with all sections, see nature.com/documents/nr-reporting-summary-flat.pdf

Life sciences study design

All studies must disclose on these points even when the disclosure is negative.

Sample size	Sample size was determined based on preliminary trials and feasibility in sample handling. Sample size was indicated in the figure legends. No statistical test was used to determine sample size.
Data exclusions	No replicates of described data were excluded for analysis.
Replication	Different independent replicates were indicated by shape whenever possible and reported in the corresponding figure legend.
Randomization	Agar-grown plants were randomized in the light chambers during the course of the experiments. For bacterial phenotypes, multiple liquid cultures from different colonies were inoculated for each experiment.
Blinding	Researchers were not fully blinded. Samples were labled by number and not treatment description during sample handling. Different researchers were involved in some repetitions.

Reporting for specific materials, systems and methods

We require information from authors about some types of materials, experimental systems and methods used in many studies. Here, indicate whether each material, system or method listed is relevant to your study. If you are not sure if a list item applies to your research, read the appropriate section before selecting a response.

Materials & experimental systems

n/a	Involved in the study	
☒	☐	Antibodies
☒	☐	Eukaryotic cell lines
☒	☐	Palaeontology and archaeology
☒	☐	Animals and other organisms
☒	☐	Clinical data
☒	☐	Dual use research of concern
☐	☒	Plants

Methods

n/a	Involved in the study	
☒	☐	ChIP-seq
☒	☐	Flow cytometry
☒	☐	MRI-based neuroimaging

Dual use research of concern

Policy information about dual use research of concern

Hazards

Could the accidental, deliberate or reckless misuse of agents or technologies generated in the work, or the application of information presented in the manuscript, pose a threat to:

No	Yes	
☒	☐	Public health
☒	☐	National security
☒	☐	Crops and/or livestock
☒	☐	Ecosystems
☒	☐	Any other significant area

Experiments of concern

Does the work involve any of these experiments of concern:

No	Yes	
☒	☐	Demonstrate how to render a vaccine ineffective
☒	☐	Confer resistance to therapeutically useful antibiotics or antiviral agents
☒	☐	Enhance the virulence of a pathogen or render a nonpathogen virulent
☒	☐	Increase transmissibility of a pathogen
☒	☐	Alter the host range of a pathogen
☒	☐	Enable evasion of diagnostic/detection modalities
☒	☐	Enable the weaponization of a biological agent or toxin
☒	☐	Any other potentially harmful combination of experiments and agents

Plants

Seed stocks	A. thaliana Col-0, rbohD (34), fls2 (SAIL_691-C4; 94), efr-1 (SALK_044334; 96) and sobir1-12 (SALK_050715; 97) and L. japonicus Gifu B-129 seeds were obtained from the institute stocks. A. thaliana pWER:FLS2-GFP (56) and pEFR:NLS-3*mVenus (79) were kindly provided by N. Geldner. The Arabidopsis efr fls2 rlp1 mutant was present at the University Tübingen, Germany (81). Numbers correspond to reference list.
Novel plant genotypes	The efr-1 fls2 sobir1-12 mutant, kindly provided by L.P. Maier and G. Felix (University Tübingen, Germany), was generated by crossing the efr-1 fls2 mutant (98) with the sobir1-12 mutant.
Authentication	*Describe any authentication procedures for each seed stock used or novel genotype generated. Describe any experiments used to assess the effect of a mutation and, where applicable, how potential secondary effects (e.g. second site T-DNA insertions, mosiacism, off-target gene editing) were examined.*
